# Photothermal-detonated functional macrophage membrane-camouflaged nano-crackers induce tumor cell wandering-anoikis

**DOI:** 10.1016/j.ajps.2026.101171

**Published:** 2026-06-04

**Authors:** Shengjie Sun, Simin Wen, Ruiqi Zhang, Yanan Fu, Huisong Hao, You Li, Yingfei Wen, Ying Huo, Yixuan Fang, Shihao Zhuang, Jia Tang, Yanglong Hou, Tianqi Wang, Meiying Wu

**Affiliations:** a School of Pharmaceutical Sciences (Shenzhen), Shenzhen Campus of Sun Yat-sen University, Shenzhen 518107, China; b School of Materials, Shenzhen Campus of Sun Yat-sen University, Shenzhen 518107, China; c The Seventh Affiliated Hospital, Sun Yat-sen University, Shenzhen 518107, China

**Keywords:** Targeting delivery, Photothermal, Macrophage membrane, Cancer-associated fibroblasts, Anoikis

## Abstract

Cancer-associated fibroblasts (CAFs) are the primary source of collagen I, which contributes to the formation of a dense tumor extracellular matrix (ECM). Non-selective targeting of collagen I through CAF inhibition may inadvertently promote tumor cell detachment and metastasis by triggering anoikis resistance. To address this, a “wandering tumor cells” strategy is proposed, combining the induction of tumor cell deadhesion with the reversal of anoikis resistance. For heterologous targeted drug delivery, thermosensitive lipids and a photosensitizer are incorporated into M1-type macrophage membranes (TMs) to enable laser-responsive activation. Based on this approach, we designed a photothermally triggered, functional macrophage membrane-camouflaged nano-cracker (TM@cP/siF-ErN) with a particle size of 162.20 ± 0.54 nm for the co-delivery of erianin (Er) and focal adhesion kinase small interfering RNA (siFAK). Er is encapsulated in anisamide (AA)-modified nanodiscs (ErN) with hydrodynamic diameter of 15.07 ± 7.24 nm to selectively inhibit collagen I synthesis in CAFs by targeting pyruvate carboxylase, thereby inducing tumor cell deadhesion. siFAK is delivered to tumor cells using cinnamaldehyde-modified polyethyleneimine (cP/siF) to formed complexes with a particle size of 98.57 ± 1.47 nm and enhance transfection efficiency, enabling effective FAK knockdown and reversal of tumor anoikis resistance. Furthermore, TMs are fragmented into debris to amplify M2-type macrophage repolarization. Experimental results show that the nano-cracker efficiently targets orthotopic 4T1 breast tumors and, upon laser-triggered detonation, releases ErN, cP/siF and M1-type macrophage membrane fragments, which collectively promote tumor anoikis by suppressing collagen I synthesis in CAFs and reversing tumor cell anoikis resistance. Moreover, it promotes the repolarization of M2-type macrophages, which synergizes with collagen I downregulation-induced infiltration of CD8⁺ T lymphocytes to enhance the antitumor immune response, collectively resulting in pronounced breast cancer suppression. This nano-cracker implements the “wandering tumor cells” strategy, offering a promising approach for improving tumor therapy and enabling heterologous targeted delivery.

## Graphical abstract

A photothermal detonated functional macrophage membrane-camouflaged nano-cracker is constructed to achieve heterologous targeting delivery. The nano-cracker induces tumor cells wandering by regulating metabolism of CAFs and promotes tumor cells anoikis.

**Figure:**
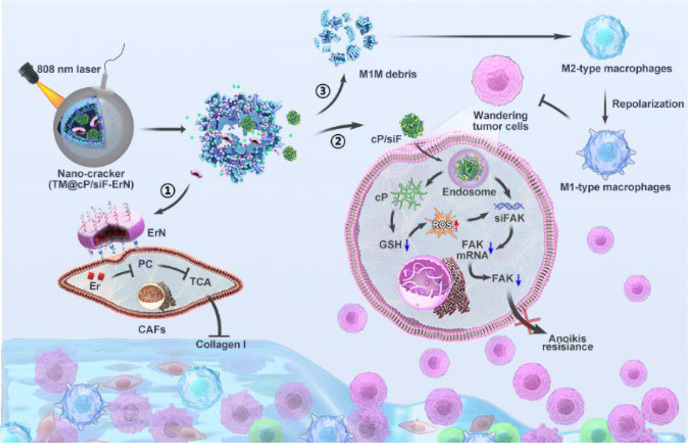

## 1 Introduction

Cancer-associated fibroblasts (CAFs), the most abundant stromal cells in the tumor microenvironment (TME), play an often underestimated role in tumor initiation, progression, and treatment resistance, particularly in solid cancers [1]. Numerous studies have demonstrated that CAFs are closely associated with the synthesis and secretion of collagen I, the primary component of the tumor extracellular matrix (ECM) [2]. The stiff, dense ECM formed by collagen I promotes adhesion between tumor cells and the matrix while simultaneously restricting immune cell infiltration [3]. Given the role of CAFs in shaping the ECM, strategies aimed at regulating the ECM by targeting CAFs—including eliminating CAF-derived TME matrix proteins, inhibiting matrix protein synthases in CAFs, directly depleting CAFs, preventing CAF activation, and reprogramming CAFs—have attracted considerable attention [[4], [5], [6], [7], [8]]. However, these approaches often indiscriminately deplete the matrix and/or matrix-producing cells, resulting in adverse effects such as accelerated tumor progression and enhanced metastasis [9]. Recent studies have shown that collagen I synthesis and secretion are mediated by the tricarboxylic acid cycle, which is regulated by pyruvate carboxylase (PC) in CAFs in specific cancers, including breast cancer, hepatocellular carcinoma, and pancreatic ductal adenocarcinoma. This pathway allows selective reduction of tumor desmoplasia without affecting ECM synthesis in normal tissues [10,11]. In these cancers, inhibiting PC activity in CAFs to reduce collagen I production may represent an effective therapeutic strategy. Erianin (Er), a novel dibenzyl compound derived from plants of the genus *Dendrobium*, has been shown to exhibit high affinity for PC and effectively inhibit its activity [12]. Accordingly, we hypothesize that Er can act as a PC inhibitor to modulate CAF metabolism, selectively decreasing collagen I secretion in specific tumor types.

Although targeting PC in CAFs can modulate collagen I synthesis to disrupt the ECM barrier, promote immune cell infiltration and enhance tumor suppression, the reduction of collagen I may also cause tumor cells to lose adhesion to stromal tissues [13]. Unlike normal cells, which typically undergo anoikis upon detachment, certain tumor cells—including breast, pancreatic and hepatocellular carcinoma cells—exhibit anoikis resistance [[14], [15], [16]]. This trait enables these tumor cells to survive in a detached, “wandering” state, facilitating metastasis and invasion [17,18]. Current studies have shown that anoikis is regulated by multiple intracellular signaling pathways, among which focal adhesion kinase (FAK), an intracellular protein tyrosine kinase, plays a key role in mediating anoikis resistance [19]. Knocking down FAK represents an effective strategy to reverse tumor cell anoikis resistance [20]. Based on this concept, we propose a “wandering tumor cells” strategy to induce tumor cell anoikis. This strategy involves two steps: first, the deadhesion of tumor cells, and second, the reversal of anoikis resistance. In the first step, adherent tumor cells are converted into wandering cells through ECM reduction, achieved by modulating CAF metabolism via a PC inhibitor. Afterward, the anoikis resistance of wandering tumor cells is reversed by silencing FAK via RNA interference (RNAi), thereby inducing cell death. The combination of these two steps triggers tumor cell anoikis, mimicking the behavior of normal cells, while simultaneously enhancing tumor therapy and promoting immune cell infiltration through ECM degradation, ultimately activating the antitumor immune response.

However, because the PC inhibitor Er and siFAK must be delivered to and act on different cell types within the TME, a heterologous targeting drug delivery system is required to achieve a synergistic effect. Cell membrane-camouflaged (CMC) delivery systems, which offer remarkable tumor-homing capability and high loading capacity, represent a promising approach [21,22]. In particular, immune cell-derived CMC systems provide excellent stealth in circulation while preserving the functional properties of the source cells. For example, M1-type macrophage membranes (M1M) can promote the repolarization of M2-type macrophages toward the M1 phenotype [23,24]. Nevertheless, most CMC delivery systems are capable of targeting only a single cell type, which seems kind of restricted considering that it involves multiple interactions between different cells and components in the process of tumor development [25]. To enable heterologous targeting, we endowed the CMC system with temperature sensitivity by incorporating thermosensitive lipids inspired by temperature-sensitive liposomes [26]. By modulating the temperature of the tumor tissue, the lipid bilayer of the temperature-sensitive CMC system becomes more fluid and reorganized, allowing controlled exposure of its cargo and enabling heterologous targeted drug delivery. To precisely control tumor tissue temperature, the photosensitizer indocyanine green (ICG) was incorporated into the lipid bilayer to enable photothermal conversion under 808 nm laser irradiation, allowing controlled disruption of the membrane and release of the loaded cargo. This thermosensitive, modified CMC delivery system provides a platform for heterologous targeted delivery, enabling synergistic therapeutic effects.

**Scheme 1:**
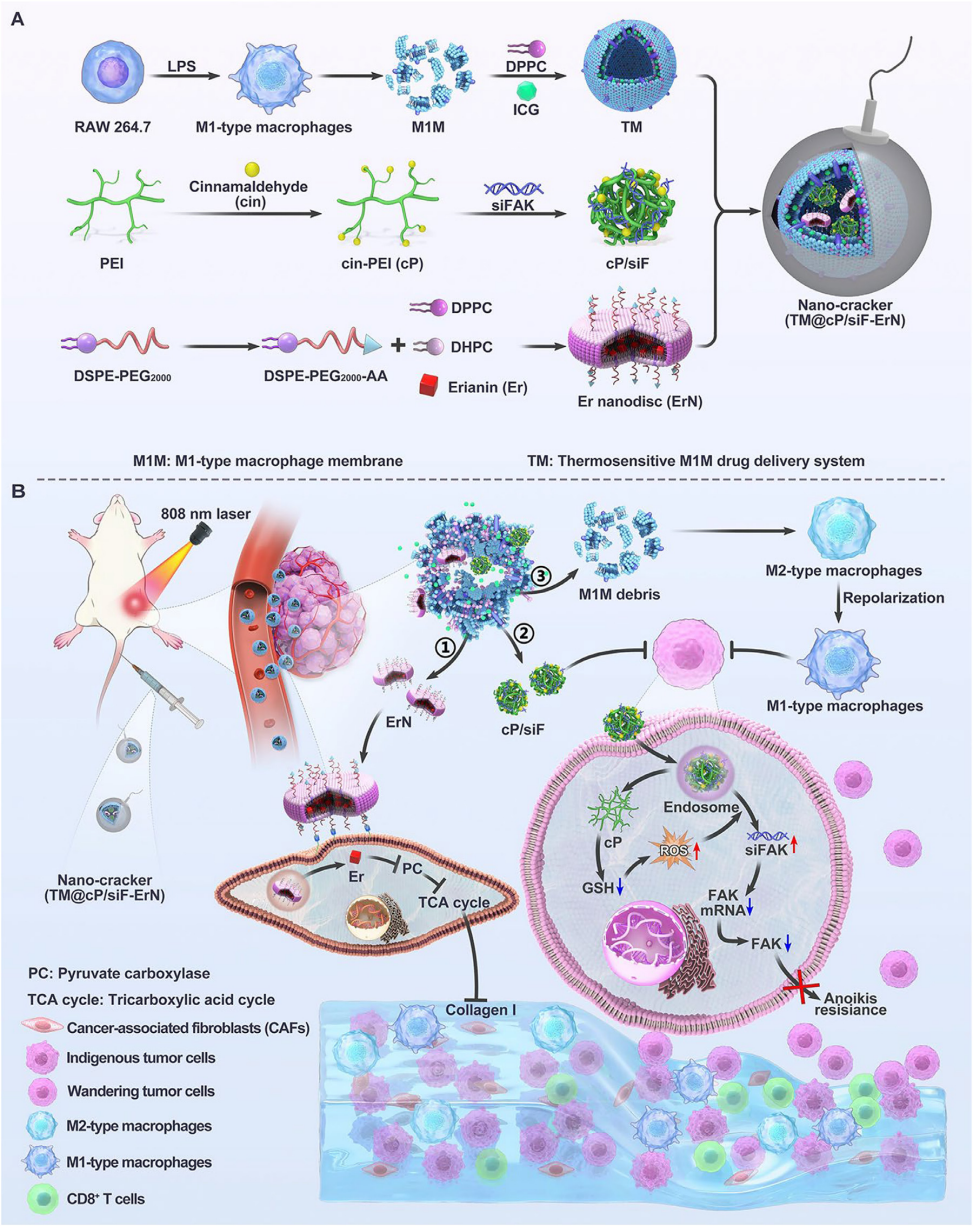
(A) Construction of nano-cracker (TM@cP/siF-ErN). (B) Photothermal detonation of nano-crackers induce wandering state and anoikis of tumor cells and enhanced immunotherapy in breast tumors.

Herein, we developed photothermally triggered, functional macrophage membrane (termed TM)-camouflaged nano-crackers for the co-delivery of Er and siFAK (Scheme 1A). Er was encapsulated in anisamide (AA)-modified lipid nanodiscs (termed ErN) to enhance CAF targeting and ensure efficient Er internalization by CAFs. Meanwhile, siFAK was complexed with cinnamaldehyde-modified polyethyleneimine (cP) (termed cP/siF) to achieve effective tumor cell uptake and transfection. By extrusion, ErN and cP/siF were encapsulated within a thermosensitive M1-type TM delivery system, forming the final nano-crackers (termed TM@cP/siF-ErN). Upon reaching the tumor tissue, the nano-crackers were triggered by 808 nm laser irradiation, causing them to disassemble into ErN, cP/siF and M1M fragments (Scheme 1B). ErN and cP/siF were selectively internalized by CAFs and tumor cells, respectively. Through the synergistic action of Er and siFAK, tumor cells were induced to enter a wandering state and subsequently undergo anoikis. Moreover, M1M fragments promoted the repolarization of M2-type macrophages toward the M1 phenotype, enhancing the antitumor immune response. Furthermore, disruption of the ECM barrier facilitated immune cell infiltration, further amplifying the antitumor immune effect and improving overall therapeutic efficacy. Thus, these nano-crackers effectively implement the “wandering tumor cells” strategy for tumor therapy and provide a versatile platform for heterologous targeted delivery of multiple drugs, offering significant potential for treating complex diseases involving interactions among diverse cell types.

## 2 Materials and methods

### 2.1 Materials

1,2-Dipalmitoyl-sn‑glycero-3-phosphocholine (DPPC) was purchased from AVT (Shanghai) Pharmaceutical Tech Co., Ltd. 1,2-Dihexanoyl-sn‑glycero-3-phosphocholine (DHPC) was obtained from Avanti Polar Lipids Inc. 1,2-Distearoyl-sn‑glycero-3-phosphoethanolamine-polyethylene glycol (DSPE-PEG_2000_) and DSPE-PEG_2000—_NH_2_ were purchased from Xi’an Ruixi Biological Technology Co., Ltd. Erianin (Er), cholesterol and indocyanine green (ICG) were obtained from Aladdin Biochemical Technology Co., Ltd. Cinnamaldehyde (cin), 4-anisic acid and coumarin 6 (C6) were purchased from Shanghai Macklin Biochemical Co., Ltd. 1-Ethyl-3-(3-dimethylaminopropyl)carbodiimide (EDC), N-hydroxysuccinimide (NHS), and polyethyleneimine (PEI, Mw ∼25,000) were obtained from Sigma-Aldrich. Hoechst 33342, 4,6-diamidino-2-phenylindole (DAPI), the Membrane and Cytosol Protein Extraction Kit, and the Cell Apoptosis Detection Kit were purchased from Beyotime Biotechnology. LysoTracker Red was obtained from Yeasen Biotechnology Co., Ltd. 1,1′-Dioctadecyl-3,3,3′,3′-tetramethylindotricarbocyanine iodide (DiR) and cyanine 5 (Cy5) were purchased from MCE. Mouse collagen I enzyme-linked immunosorbent assay (ELISA) kit was purchased from Jiangsu Meimian Industrial Co., Ltd. PC Activity Assay Kit, and mouse TNF-α, iNOS, TGF-β1 and Arg1 ELISA kits were obtained from Beijing Solarbio Science & Technology Co., Ltd. siFAK (5′-CTATAAAGCTTCCAAAGGA-3′, sense), negative control siRNA (siN), carboxyfluorescein (FAM)-labeled siFAK (FAM-siF), and primers were purchased from Guangzhou Ribo Bio-Technology. Antibodies used in this study are listed in Table S1.

### 2.2 Synthesis of cP

cP was synthesized via a Schiff base reaction. Briefly, 2.0 ml cin (12.6 µg) in dimethyl sulfoxide (DMSO) was added dropwise to 2.0 mL PEI (50.0 µg) in DMSO and stirred overnight at 45 °C (318.15 K) under an argon atmosphere. The resulting mixture was then dialyzed against ultrapure water for 2 d and subsequently freeze-dried. The structure of cP was confirmed by proton nuclear magnetic resonance (^1^H NMR) spectroscopy using a Bruker 400 MHz spectrometer in D_2_O at room temperature.

### 2.3 In vitro GSH depletion

The *in vitro* glutathione (GSH)-depletion ability of cP was evaluated using a 5,5′-dithiobis-(2-nitrobenzoic acid) (DTNB) assay. Phosphate-buffered saline (PBS), PEI, cin and cP were each incubated with GSH solution for various times (0, 0.5, 1, 2 and 4 h) at 37 °C (310.15 K). Subsequently, an equal amount of DTNB was added, and the absorbance of the mixture at 412 nm was measured immediately.

### 2.4 Preparation of cP/siF

siFAK (1.0 mg/ml) and cP (1.4 mg/ml) were each dissolved separately in RNase-free water. The siFAK solution was then added dropwise to the cP solution in equal volume and incubated at room temperature for 30 min to form cP/siF. Similarly, complexing cP with siN or FAM-siF produced cP/siN and cP/FAM-siF, respectively. P/siF was prepared using the same procedure.

### 2.5 Agarose gel electrophoresis retardation assay

cP/siF complexes with various N/P ratios were prepared using different concentrations of PEI while keeping the siFAK amount constant (0.3 µg). Then, 2.5 µl of 5 × loading buffer was mixed with 10 µl free siFAK or 10 µl cP/siF complexes at different N/P ratios, and the samples were electrophoresed on a 1% agarose gel in Tris-acetate EDTA (TAE) buffer at 90 V for 30 min. RNA bands were visualized using the Essential V6 imaging platform (UVITEC Cambridge).

### 2.6 Synthesis of DSPE-PEG_2000_-AA

DSPE-PEG_2000_-AA was synthesized for CAF targeting. Briefly, 4-anisic acid (41.0 mg, 0.27 mmol) and NHS (46.0 mg, 0.4 mmol) were dissolved in 4.0 mL methanol and stirred at 0 °C (273.15 K). EDC (62.0 mg, 0.4 mmol) in 4.0 mL methanol was added dropwise, and the mixture was stirred at 0 °C (273.15 K) for 0.5 h. Then, 500 µL triethylamine (TEA) was added to adjust the solution to a slightly basic pH. DSPE-PEG_2000__-__—_NH_2_ (41.0 mg, 0.27 mmol) in methanol was added under stirring, and the reaction mixture was gently heated to 40 °C (313.15 K) for 2 h. After completion, the solvent was removed using a rotary evaporator, and the residue was redissolved in dichloromethane. The solution was subjected to liquid-liquid extraction with 0.1 M HCl three times, followed by extraction with a saturated sodium chloride solution three times, and then dried over anhydrous sodium sulfate. The solids were removed by filtration, and the filtrate was evaporated. DSPE-PEG_2000_-AA was obtained after vacuum drying overnight. The structure of DSPE-PEG_2000_-AA was confirmed by ^1^H NMR spectroscopy in *d6*-DMSO.

### 2.7 Preparation of ErN

DPPC, DHPC and DSPE-PEG_2000_-AA were dissolved in 4.0 ml chloroform at a molar ratio of 3:1:0.5, while erianin was dissolved in 1.0 ml methanol. ErN were prepared using the thin-film hydration method. The organic solvents were removed by rotary evaporation to form a lipid film over 1 h. Subsequently, 1 ml deionized water was added, and the mixture was rotated to hydrate the film at 45 °C (318.15 K) for 30 min. The resulting suspension was sonicated using a probe sonicator for 3 min to obtain ErN. Finally, the nanodiscs were sequentially filtered through 450 nm and 220 nm syringe filters and stored at 4 °C (277.15 K). Similarly, nanodiscs loaded with DiR or Cy5 were prepared to obtain DiRN or Cy5N, and blank nanodiscs (N) were prepared using the same procedure.

### 2.8 Repolarization of RAW 264.7 cells and extraction of M1M

The M1M was extracted following the instructions of the Membrane and Cytosol Protein Extraction Kit. RAW 264.7 cells were cultured to high density and polarized to M1-type macrophages using lipopolysaccharide (LPS, 100 ng/ml). The M1 macrophages were then collected and lysed using the freeze-thaw method. Cell membrane fragments were isolated by high-speed centrifugation and stored at −80 °C (193.15 K).

### 2.9 Preparation and characterization of nano-crackers TM@cP/siF-ErN

DPPC (20.0 mg) and cholesterol (5.0 mg) were dissolved in 4.0 ml chloroform, and ICG (200.0 µg) was dissolved in 1.0 ml methanol. Blank nano-crackers were prepared using the thin-film hydration method. The organic solvents were removed by rotary evaporation to form a lipid film for 1 h in the dark. Subsequently, 1.0 ml deionized water containing M1M was added, and the mixture was rotated to hydrate the film for 30 min in the dark. cP/siF and ErN were then co-loaded, and the nano-crackers (TM@cP/siF-ErN) were formed by extrusion through a 220 nm syringe filter using an Avanti liposome extruder. The particle size and zeta potential of the nano-crackers were measured using a Malvern particle sizer, and their morphology was examined by transmission electron microscopy (TEM). Similarly, TM loaded only with cP/siF and N (TM@cP/siF-N) or only with cP/siN and ErN (TM@cP/siN-ErN) were prepared using the same procedure.

### 2.10 Photothermal-responsive rupture of nano-crackers

Nano-crackers were prepared at a concentration of 100 µg/ml of ICG. The solution was subjected to laser irradiation at 1.0 W/cm^2^, causing the temperature to rise to 45 °C (318.15 K), after which the laser was immediately turned off to allow the temperature to return to 37 °C (310.15 K). This heating-cooling cycle was repeated five times, and the temperature-time curve was recorded. Finally, the nano-cracker solution was diluted to measure particle size using a Malvern particle sizer and to observe morphology by TEM.

### 2.11 SDS-PAGE of M1M protein

Membrane proteins from RAW 264.7 cells were extracted using RIPA lysis buffer and quantified with a bicinchoninic acid (BCA) kit. Proteins from M1-type macrophages (M1), M1M and TM were then analyzed by sodium dodecyl sulfate-polyacrylamide gel electrophoresis (SDS-PAGE), with protein amounts standardized across samples. Electrophoresis was performed at 75 V for 30 min, followed by 150 V for 1 h at room temperature. The gels were stained with Coomassie Brilliant Blue for 30 min, washed three times, and subsequently scanned and analyzed using image analysis software.

### 2.12 Cell culture

Mouse breast tumor cells (4T1) and mouse macrophage cells (RAW 264.7) were obtained from the National Collection of Authenticated Cell Cultures, and mouse fibroblasts (3T3) were purchased from Procell Life Science & Technology Co., Ltd. 4T1 and RAW 264.7 cells were cultured in Dulbecco’s modified Eagle’s medium (DMEM) supplemented with 10% fetal bovine serum (FBS) and 1% antibiotic solution (100 U/ml penicillin and 100 µg/ml streptomycin) at 37 °C (310.15 K) in a humidified incubator with 5% CO_2_ (Thermo Fisher Scientific Inc., Thermo 3111, Rochester, NY, USA). 3T3 cells were cultured in DMEM supplemented with 10% calf serum and 1% antibiotic solution under the same conditions.

### 2.13 Intracellular uptake and lysosomal escape

To evaluate cellular uptake, 4T1 cells were seeded in 6-well plates at a density of 2 × 10^5^ cells/well and allowed to adhere overnight. Cells were then treated with cP/FAM-siF complexes (containing 1.5 µg/ml FAM-labeled siRNA) for varying times (0, 0.5, 1, 2 and 4 h). After incubation, cells were washed three times with PBS and resuspended in flow cytometry (FCM) staining buffer. Cellular internalization of cP/FAM-siF was quantified by FCM (CytoFLEX, Beckman Coulter, USA).

To investigate the lysosomal escape of cP/FAM-siF, 4T1 cells were seeded in 6-well plates at a density of 2 × 10^5^ cells/well and incubated with cP/FAM-siF for 2 or 8 h. The cells were then washed three times with PBS to remove residual materials. For subcellular localization, lysosomes were stained with LysoTracker Red for 15 min, and nuclei were stained with Hoechst 33342 for 15 min. Finally, the samples were imaged in detail using a confocal laser scan microscope (CLSM).

Similarly, C6 was used in place of erianin to evaluate the cellular uptake of ErN in 3T3 cells. Cells were seeded in 6-well plates at a density of 2 × 10^5^ cells/well and allowed to adhere for 12 h, then incubated with C6-loaded nanodiscs (C6N) for 0, 1, 2 or 4 h. After incubation, the cells were washed three times with PBS and resuspended in FCM staining buffer. Cellular uptake of C6N was visualized and quantified by FCM.

### 2.14 Intracellular GSH depletion

To assess intracellular GSH depletion, 4T1 cells were seeded in 6-well plates at a density of 2 × 10^5^ cells/well and incubated overnight. The cells were then treated with different formulations (PBS, P/siF, cP/siF), collected by trypsinization, and centrifuged at 1000 × g for 5 min. The cell pellets were stained with ThiolTracker™ Violet fluorescent probe at 37 °C (310.15 K) for 30 min, washed three times with PBS, and resuspended in staining buffer. Fluorescence intensity was measured by FCM.

### 2.15 Cell viability of 4T1 cells after siFAK treatment

4T1 cells were seeded in 96-well plates at a density of 5 × 10^3^ cells/well and allowed to adhere for 12 h. The cells were then treated with media containing different formulations at various siRNA concentrations (0.02, 0.04, 0.16, 0.75, 1.5 µg/ml). After 24 h, cell viability was assessed using the CCK-8 assay.

### 2.16 qPCR analysis of gene expression in 4T1 cells

4T1 cells were seeded in 6-well plates at a density of 2 × 10^5^ cells/well and allowed to adhere for 12 h. The cells were then treated with different formulations—PBS (control), cP/siN, P/siF and cP/siF—each containing siRNA at 1.25 µg/ml for 24 h. Total RNA was extracted using RNAiso Plus reagent according to the manufacturer’s instructions. Reverse transcription (RT) was performed with an RT reagent kit that included a genomic DNA removal step to ensure pure cDNA synthesis. Quantitative polymerase chain reaction (qPCR) was conducted using the PerfectStart Green qPCR SuperMix Kit on a Roche LightCycler 96 system, with β-actin serving as the endogenous reference gene for normalization of gene expression.

### 2.17 Anoikis efficacy in 4T1 cells

Two models were established using 6-well plates: a normal-adherent model and a low-adherent model. For the low-adherent model, plates were pretreated with poly-hydroxyethyl methacrylate (poly-HEMA). 4T1 cells were seeded at a density of 2 × 10^5^ cells/well and allowed to adhere for 12 h. The cells were then treated with PBS, cP/siN, or cP/siF (1.25 µg/ml siRNA) for 24 h and subsequently collected. The anoikis rate was determined by staining with the Cell Apoptosis Detection Kit and analyzed by FCM.

### 2.18 Intracellular PC activity and collagen Ⅰ secretion in 3T3 cells

Intracellular PC activity was measured using a PC Activity Assay Kit, and total intracellular and extracellular collagen I levels were quantified with a mouse collagen I ELISA kit. 3T3 cells were seeded in 6-well plates at a density of 2 × 10^5^ cells/well and allowed to adhere for 12 h, then treated with PBS, Er, blank nanodiscs (N) or ErN for 24 h. After treatment, the cells were collected and processed according to the respective assay kit protocols.

### 2.19 Macrophage polarization

FCM was used to analyze the polarization of bone marrow-derived macrophages (BMDMs). BMDMs were isolated from BALB/c mice and seeded in 6-well plates at a density of 1 × 10^5^ cells/well, then cultured for 12 h. The culture medium was subsequently replaced with fresh medium containing either LPS (100 ng/ml) to induce M1 polarization or IL-4 (40 ng/ml) to induce M2 polarization. M1-type macrophages were used for membrane extraction, while M2-type macrophages were used in repolarization experiments.

M2-type macrophages were treated with different formulations, with the LPS-treated group serving as the positive control. The cells were cultured for an additional 24 h, then washed with PBS and collected using a cell scraper. After centrifugation, the cells were resuspended in 300 µl PBS. For surface marker staining, cells were incubated with PE/Cyanine 7-anti-CD11b and FITC-anti-CD86 antibodies (BioLegend, USA) for 1 h on ice in staining buffer. After three washes, the cells were fixed and permeabilized for intracellular staining with APC-anti-CD206. Following a final wash, the cells were collected for analysis. Data were acquired using a CytoFLEX flow cytometer and analyzed with FlowJo software.

### 2.20 Co-culture of 4T1 and 3T3 cells in a 3D model

A Corning® 96-well Black/Clear Round Bottom Ultra-Low Attachment Spheroid Microplate was used for the 3D co-culture. 4T1 and 3T3 cells were seeded at a 1:1 ratio with a total density of 2 × 10^5^ cells/well. The complete culture medium consisted of DMEM supplemented with 10% FBS, 100 U/ml penicillin, 100 µg/ml streptomycin, and 10 mg/ml collagen I. After 12 h, cells settled and aggregated at the bottom of the wells. Continued culture for an additional 2 days resulted in the formation of a single, uniform mixed-cell spheroid at the center of each well. Once spheroids were established, they were treated for 24 h with PBS, the mixed group cP/siF + ErN, TM@cP/siF-N + L, TM@cP/siN-ErN + L, TM@cP/siF-ErN or TM@cP/siF-ErN + L (siRNA dose: 1 µg/ml). The three laser-irradiated groups were exposed to 808 nm laser irradiation at 1.0 W/cm^2^ for 5 min prior to drug treatment to maintain a local temperature of 37–45 °C (310.15–318.15 K). After treatment, the spheroids were then imaged using confocal laser scanning microscopy with Z-stack acquisition. The cells were collected to assess collagen I levels and 4T1 cell apoptosis.

### 2.21 Animal model

Female BALB/c mice aged 6–8 weeks were obtained from the Guangdong Medical Laboratory Animal Center (Foshan, China). All animal experiments were conducted in accordance with the ethical guidelines approved by the Institutional Animal Care and Use Committee of Sun Yat-sen University (Shenzhen, China; Approval No SYSU-IACUC-2024-000619). To establish unilateral tumor models, 4T1 cells (1 × 10^6^ cells suspended in a PBS-Matrigel mixture) were subcutaneously injected into the left flank of each mouse. Tumor growth was monitored until volumes reached approximately 120 mm^3^, at which point the mice were randomly assigned to experimental groups.

### 2.22 Biodistribution in vivo and *ex vivo*

*In vivo* fluorescence imaging and biodistribution were evaluated in 4T1 tumor-bearing mice (*n* = 3 per group). Following intravenous injection of different formulations (0.5 mg/kg DiR per mouse), mice were imaged at 0, 2, 4, 8, 12, 24 and 36 h using an *in vivo* imaging system (IVIS, Bruker, FX Pro, USA; Ex/Em = 740 nm/790 nm). Afterward, the main organs (heart, liver, spleen, lungs and kidneys) and tumors were harvested post-mortem and imaged *ex vivo* using IVIS.

### 2.23 Photothermal-responsive rupture and heterologous targeting of nano-crackers in tumor tissue in vivo

*In vivo* drug targeting and release from blood vessels were evaluated in 4T1 tumor-bearing mice (*n* = 3 per group). Mice were intravenously injected with different formulations (1 mg/kg FAM-siF and 0.5 mg/kg DiR per mouse) and exposed to laser irradiation at 42 °C (315.15 K) for 5 min. Tumor tissues were collected at 2, 4 and 8 h post-injection, including an additional 8 h group with laser treatment, and subjected to immunofluorescence staining using Alexa Fluor® 594-anti-CD31, IF 546-anti-α-SMA, and DAPI.

### 2.24 In vivo antitumor efficacy

Mice with unilateral tumors were used to evaluate tumor growth inhibition. The animals were randomly assigned to groups (*n* = 5 per group) and treated via tail vein injection on Day 1, 4 and 7 with the following formulations: PBS, cP/siF + ErN, TM@cP/siF-N + L, TM@cP/siN-ErN + L, TM@cP/siF-ErN, or TM@cP/siF-ErN + L (siRNA dose: 1 mg/kg; Er dose: 25 mg/kg). For the three laser-treated groups, 808 nm laser irradiation (1.0 W/cm^2^, 37–45 °C) was applied for 5 min at 8 h post-injection. Body weight and tumor dimensions were measured every other day throughout the study. Tumor volumes (V) were calculated using the formula V = 0.5 × L × W^2^, where L is the longest tumor axis, and W is the perpendicular shortest diameter. On Day 14 post-treatment, mice were euthanized, and major organs (heart, liver, spleen, lungs and kidneys) along with portions of tumor tissue were collected for hematoxylin and eosin (H&E) staining and immunofluorescence analysis.

Selected tumor specimens were mechanically dissociated, filtered through 70 µm nylon mesh strainers, and processed for flow cytometric analysis. To assess macrophage repolarization, tumor-derived cells were incubated with PE/Cyanine 7-anti-CD11b and FITC-anti-CD86 antibodies for 1 h on ice in staining buffer. After three washes, the cells were fixed and permeabilized for intracellular labeling with APC-anti-CD206. Following a final wash, cells were resuspended for FCM. For T-cell infiltration analysis, tumor-derived cells were incubated with PE/Cyanine 7-anti-CD3, FITC-anti-CD8a, and APC-anti-CD4 antibodies (BioLegend, USA) for 1 h on ice, washed three times, and resuspended for detection. Furthermore, tumor tissues were homogenized, and the supernatants were collected to quantify TGF-β1, TNF-α, Arg1 and iNOS levels using ELISA kits.

### 2.25 Statistical analysis

Quantitative data are presented as mean ± standard deviation (SD). Statistical significance for pairwise comparisons was determined using Student’s *t*-test, while differences among three or more groups were analyzed by one-way ANOVA. Significance levels were defined as *P < 0.05, **P < 0.01, and ***P < 0.001, with nonsignificant differences indicated as ns.

## 3 Results and discussion

### 3.1 Preparation and characterization of TM@cP/siF-ErN

**Fig. 1:**
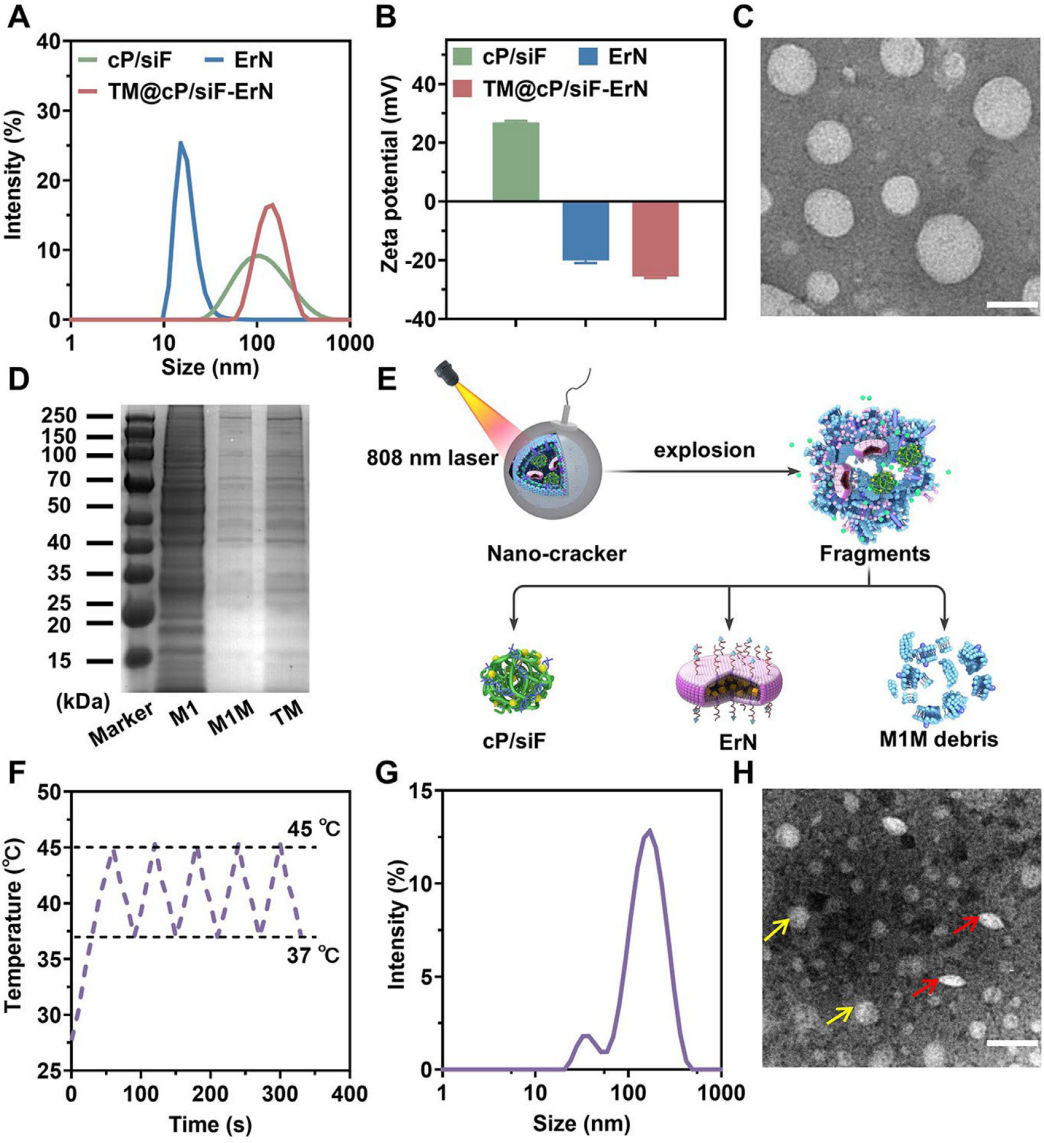
Characterization of the nano-cracker TM@cP/siF-ErN. (A) Hydrodynamic diameters and (B) zeta potentials of cP/siF, ErN and TM@cP/siF-ErN. Data are presented as mean ± SD (*n* = 3); (C) TEM image of TM@cP/siF-ErN. Scale bar: 100 nm; (D) SDS-PAGE gel electrophoresis analysis of proteins presented on M1, M1M and thermosensitive M1M drug delivery system (TM); (E) Schematic illustration of the explosion of nano-crackers after laser irradiation; (F) Photothermal conversion ability of TM@cP/siF-ErN solution for five laser on/off cycles with an 808 nm laser irradiation (1.0 W/cm^2^) at a mild temperature range; (G) Hydrodynamic diameters of TM@cP/siF-ErN after laser irradiation; (H) TEM image of TM@cP/siF-ErN after laser. Scale bar: 100 nm. Red arrows: ErN. Yellow arrows: cP/siF.

To simultaneously regulate tumor cells and CAFs, an innovative photothermal functional macrophage membrane-camouflaged nano-cracker was developed to co-deliver siFAK and Er, with each loaded into distinct nanoparticles for heterologous targeting drug delivery. Previous studies have shown that GSH depletion can enhance gene transfection in tumor cells [27]. To achieve high transfection efficiency, the cationic polymer PEI was pre-modified with cin to deplete the increased GSH levels in tumor cells. The resulting cP was successfully synthesized via a Schiff base reaction (Fig. S1) and characterized by ^1^H NMR spectroscopy, confirming successful modification with an 8% grafting rate (Fig. S2). As illustrated in Fig. S3, cP exhibited excellent GSH depletion capability. Then, cP/siF was prepared through electrostatic adsorption. The results demonstrated that the complexes at an N/P ratio of 10:1 exhibited efficient loading (Fig. S4), with a uniform particle size of 98.57 ± 1.47 nm, a polydispersity index (PDI) of 0.121 ± 0.017 (Fig. 1A), and a zeta potential of 27.64 ± 2.83 mV (Fig. 1B). The low PDI indicates that the measured average particle size reliably represents the majority of cP/siF particles. The pronounced positive surface charge facilitates selective uptake by tumor cells, which typically have higher negative membrane charge densities [28]. TEM images (Fig. S5) further confirmed the spherical morphology and uniform size distribution of the complexes. Agarose gel electrophoresis results (Fig. S4) confirmed that siFAK was completely complexed by cP, with a loading content of approximately 36.36%. Er, a PC inhibitor, was loaded into nanodiscs for selective targeting of CAFs to minimize off-target effects on other cells. DSPE-PEG_2000_-AA, which possesses strong CAF-targeting capability, was successfully synthesized following a reported method and incorporated into the nanodiscs (Figs. S6 and S7) [29]. The resulting ErN exhibited a small hydrodynamic diameter of 15.07 ± 7.24 nm, a PDI of 0.482 ± 0.007 (Fig. 1A), and a zeta potential of −20.20 ± 0.68 mV (Fig. 1B). TEM images (Fig. S8) revealed that ErN exhibited a disc-like morphology with a lateral dimension of approximately 25–40 nm and a short-axis diameter of 15–20 nm. This oblate structure not only facilitates diffusion within the dense tumor ECM but also, combined with CAF-targeted modification, enhances cellular internalization by CAFs [30,31]. Quantitative analysis by high-performance liquid chromatography indicated that ErN achieved an encapsulation efficiency of 86% and a drug loading content of 1.6% for Er. Subsequently, the TM was prepared using the thin-film hydration method. Thermosensitive lipid DPPC and the photosensitizer ICG were incorporated into the membrane to confer controllable photothermal responsiveness, enabling efficient heterologous targeting delivery.

The nano-cracker was prepared by extruding TM with cP/siF and ErN, resulting in well-dispersed particles with a hydrodynamic diameter of 162.20 ± 0.54 nm, a PDI of 0.215 ± 0.019, and a spherical morphology (Fig. 1A and 1C), supporting prolonged systemic circulation and efficient passive tumor targeting [32]. The negative zeta potential (−24.89 ± 1.26 mV) confirmed successful TM coating (Fig. 1B). SDS-PAGE analysis verified the presence and integrity of the M1M (Fig. 1D), with TM protein bands consistent with those of M1M, indicating that the membrane proteins retained their physiological functionality. To confirm the co-encapsulation of cP/siF and ErN within TM, each component was fluorescently labeled, and their co-localization was evaluated by FCM (Fig. S9). Compared with TM@cP/siF-N, TM@cP/Cy5-siF-C6N showed a high double-positive fluorescence rate of 98.2%, indicating efficient co-localization of cP/Cy5-siF and C6N. These results demonstrate that the employed method enables effective co-encapsulation of cP/siF and ErN within the TM carrier. Furthermore, the nano-cracker exhibited excellent stability, as indicated by consistent particle size distribution and PDI in PBS over 7 days, with no significant fluctuations (Fig. S10). With the incorporation of DPPC and ICG, the nano-crackers could be triggered by laser irradiation to disassemble into cP/siF, ErN, and M1M debris (Fig. 1E). To evaluate their photothermal conversion capability, TM@cP/siF-ErN was irradiated with an 808 nm laser, and temperature changes were monitored using a thermal infrared imager. The temperature was maintained within a mild range of 37–45 °C (310.15–318.15 K) to match the phase transition temperature of DPPC, which occurs at 42 °C (315.15 K, Fig. 1F). Considering that localized laser irradiation is applied to tumor sites *in vivo*, we first confirmed that irradiation at 1.0 W/cm^2^ does not generate reactive oxygen species (ROS) or induce cytotoxicity in tumor cells (Figs. S11 and S12), as the local temperature is maintained within 37–45 °C (310.15–318.15 K) for 5 min. After laser irradiation, multiple peaks in the particle size distribution confirmed the rupture of the nano-cracker (Fig. 1G). Furthermore, TEM images revealed the re-emergence of particles with distinct sizes and morphologies. As illustrated in Fig. 1H, red arrows indicate disc-shaped ErN with diameters of 20–50 nm, while yellow arrows mark spherical cP/siF particles with diameters of 80–100 nm. These observations demonstrate the successful exposure of cP/siF and ErN after laser irradiation, enabling subsequent heterologous targeting delivery. We further evaluated the release profile of Er from TM@cP/siF-ErN following laser treatment. As illustrated in Fig. S13, under acidic conditions mimicking the lysosomal environment, ErN released from TM@cP/siF-ErN after laser irradiation exhibited a release pattern comparable to that of non-encapsulated ErN. These results further confirm the successful release of both cP/siF and ErN upon laser irradiation, with the photothermal-triggered disassembly of ErN enabling efficient Er release.

### 3.2 Inhibition of collagen I synthesis and reversion of anoikis resistance

**Fig. 2:**
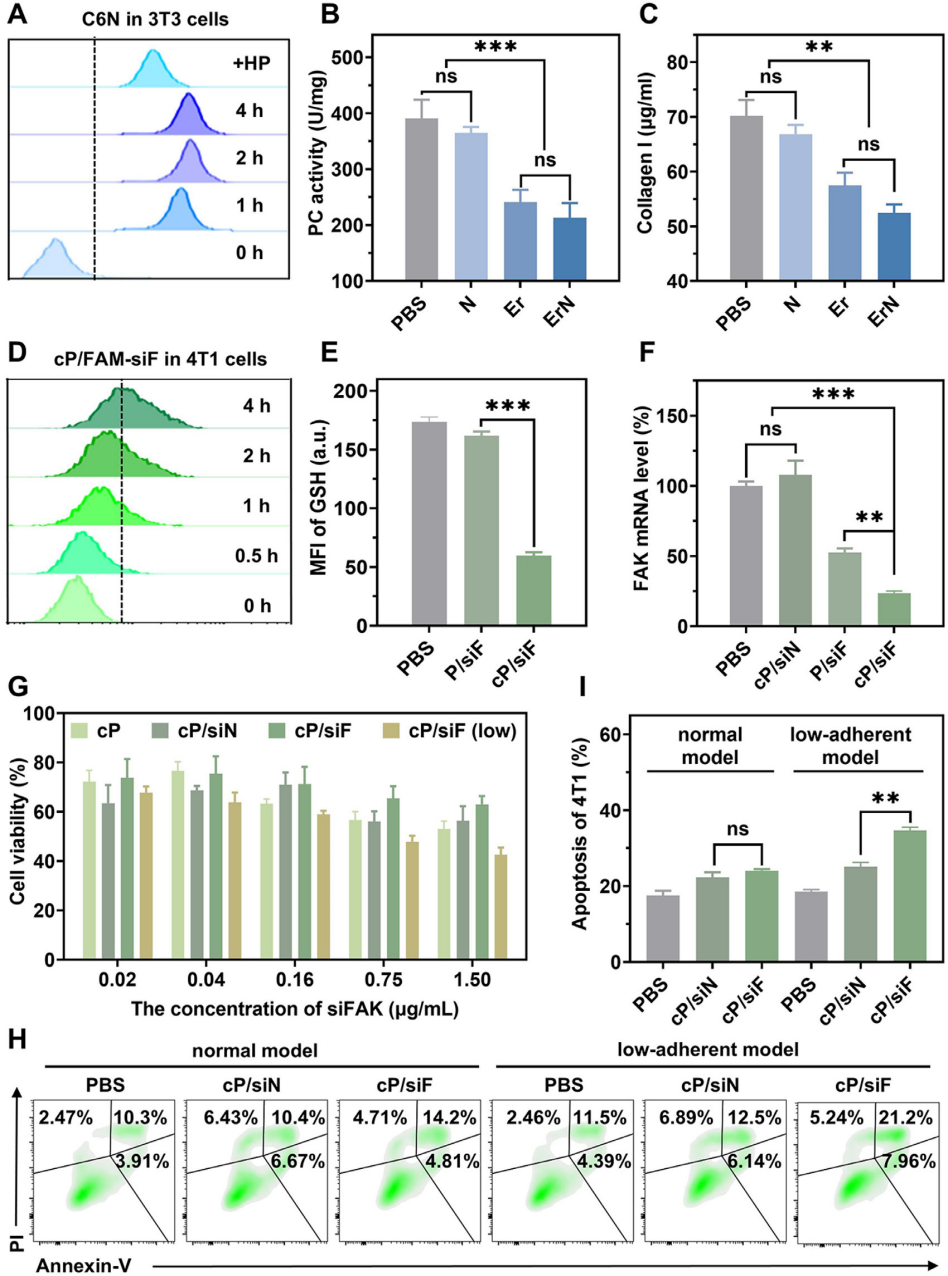
*In vitro* cellular effect of ErN and cP/siF on 3T3 and 4T1 cells, respectively. (A) FCM analysis of C6N uptake by 3T3 cells at different times. HP: incubation with 3T3 cells for 1 h before C6N treatment; (B) PC activity of 3T3 cells (*n* = 3); (C) Extracellular and intracellular collagen I concentration of 3T3 cells (*n* = 3); (D) FCM analysis of cP/FAM-siF uptake by 4T1 cells at different times; (E) Flow cytometric quantitative analysis of intracellular GSH in 4T1 cells (*n* = 3); (F) FAK mRNA level in 4T1 cells analyzed by qPCR (*n* = 3); (G) Cell viability of 4T1 cells in normal model and low-adherent model (low) (*n* = 3); (H) FCM assay and (I) quantitative analysis of the apoptosis of 4T1 cells in normal model and low-adherent model with different treatments (*n* = 3). Data are presented as mean ± SD.

After laser irradiation, ErN and cP/siF were internalized by CAFs and tumor cells, respectively. To evaluate CAF targeting, C6 was used as a fluorescent substitute for Er to prepare C6N. Owing to the modification of the targeting ligand AA, C6N was rapidly internalized by 3T3 cells within 1 h (Fig. 2A). Moreover, pre-incubation with haloperidol (HP), a competitive inhibitor of AA, significantly reduced the uptake of C6N by 3T3 cells. This competitive inhibition study demonstrated that C6N targets the sigma-1 receptor on CAFs and is internalized via AA-mediated endocytosis [33]. After internalization by CAFs, erianin was released from the nanodiscs into the cytoplasm and functioned as a PC inhibitor, significantly reducing PC activity from 390.8 ± 33.4 U/mg to 213.0 ± 26.4 U/mg (Fig. 2B). Subsequently, inhibition of PC, a key enzyme in cellular biosynthesis, led to a marked decrease in collagen I production, from 70.2 ± 2.9 µg/ml to 52.5 ± 1.6 µg/ml (Fig. 2C). These results demonstrate that ErN effectively targets 3T3 cells and suppresses collagen I synthesis through PC inhibition.

Meanwhile, cP/siF was internalized by 4T1 cells to reverse anoikis resistance. FAM-siF was employed to evaluate the cellular uptake of cP/FAM-siF in 4T1 cells. As illustrated in Figs. 2D and S14, cP/FAM-siF was rapidly internalized in a time-dependent manner within 4 h, indicating that cinnamaldehyde modification of PEI did not impair endocytic efficiency. Following internalization, cP/FAM-siF localized to lysosomes at 2 h and subsequently escaped into the cytoplasm by 8 h, attributable to the proton sponge effect of the amine groups on cP (Fig. S15) [34]. In addition, cP/siF significantly reduced intracellular GSH levels due to cinnamaldehyde modification (*P* < 0.001) (Fig. 2E). It has been demonstrated that GSH depletion can promote the generation of ROS, thereby enhancing lysosomal escape and improving siRNA transfection efficiency [27]. Subsequently, siFAK-mediated silencing of the FAK gene was evaluated by qPCR. As illustrated in Fig. 2F, no significant difference in intracellular FAK mRNA levels was observed between the PBS group and the cP/siN group, indicating that GSH depletion induced by cP alone did not affect FAK gene expression. In contrast, the FAK silencing efficiency of the cP/siF group was 26.7% higher than that of the P/siF group, confirming that cin modification significantly enhanced transfection efficiency (*P* = 0.0020). FAK has been established as a critical regulator of anchorage-independent tumor cell growth and anoikis resistance, and its expression is closely associated with breast tumor progression and metastasis [19]. To verify whether downregulation of FAK expression could reverse anoikis resistance, normal-adherent and low-adherent cell models were established to evaluate the cytotoxicity and apoptosis of 4T1 cells following different treatments. As illustrated in Fig. 2G, cP/siF exhibited higher cytotoxicity in the low-adherent model than in the normal-adherent model. To further assess apoptosis, 4T1 cells were analyzed by FCM. After treatment with cP/siF, the apoptosis rate in the low-adherent model significantly increased to 34.7% ± 0.8% (*P* = 0.0091) (Fig. 2H and 2I). In contrast, no significant difference was observed between the cP/siN and cP/siF groups in the normal-adherent model, indicating that inhibition of the FAK signaling pathway specifically induces anoikis in low-adherent tumor cells.

### 3.3 Macrophage repolarization and anoikis induced by TM@cP/siF-ErN

**Fig. 3:**
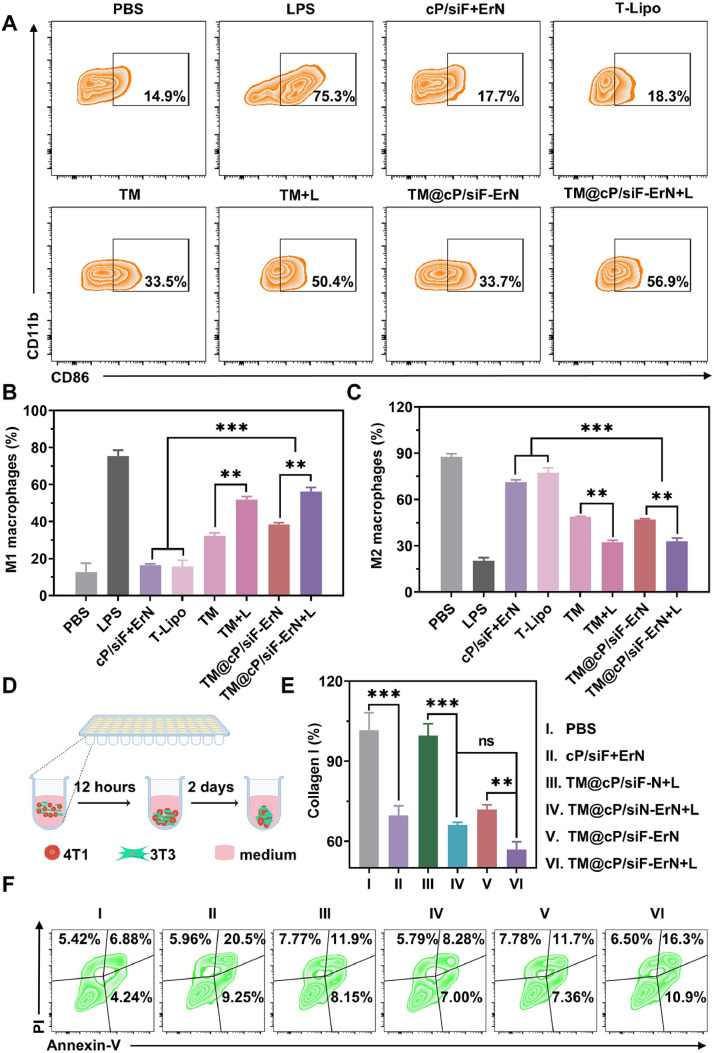
Nano-crackers induced macrophage repolarization *in vitro* and anoikis in 3D spheroid model of 4T1 and 3T3 cells. (A–C) FCM analysis of M1-type macrophages labeled with anti-CD86 antibody (A) and the quantitative analysis of M1-type macrophages (B) and M2-type macrophages (C) (*n* = 3); (D) Diagram of the establishment of 3D spheroid model of 4T1 and 3T3 cells at a ratio of 1:1; (E) Total collagen I quantified by ELISA (*n* = 3); (F) Apoptosis rate of 4T1 cells in 3D spheroid model by FCM analysis. Data are presented as mean ± SD.

The detonation of the nano-crackers not only exposed ErN and cP/siF for heterologous targeting but also led to the fragmentation of M1M, generating membrane debris that could promote the repolarization of M2-type macrophages toward the M1 phenotype. The repolarization effect was evaluated by FCM, with LPS and thermosensitive liposomes (T-Lipo) used as control groups. As illustrated in Figs. 3A–3C and S16, neither the cP/siF + ErN mixture nor T-Lipo induced M2-to-M1 macrophage polarization. In contrast, treatment with TM and TM@cP/siF-ErN increased the proportion of M1-type macrophages to 32.3% ± 1.6% and 38.4% ± 1.0%, respectively, accompanied by a reduction in the M2-type macrophage population to 48.8% ± 0.5% and 47.0% ± 0.6%. These results indicate that the macrophage repolarization effect of TM@cP/siF-ErN is mainly mediated by the presence of M1M within the TM system. Meanwhile, upon laser pre-irradiation at 808 nm, both TM and TM@cP/siF-ErN treatments exhibited a markedly enhanced repolarization effect, with the proportions of M2-type macrophages decreasing to 32.1% ± 1.4% and 32.8% ± 2.2%, and the proportions of M1-type macrophages increasing to 51.8% ± 1.7% and 56.2% ± 2.3%, respectively. This enhancement is likely attributable to the laser-induced fragmentation of the M1M, whereby the resulting membrane debris could interact with a larger population of M2-type macrophages, thereby amplifying the repolarization effect.

As the nano-cracker is designed to act on two distinct cell types to implement the “wandering tumor cells” strategy, a conventional 2D cell model can only independently demonstrate the inhibition of collagen I synthesis in CAFs or the reversal of anoikis resistance in tumor cells. To further validate the anoikis-inducing effect of the nano-cracker, a 3D cell spheroid model was established, as illustrated in Fig. 3D. 4T1 and 3T3 cells were co-cultured at a 1:1 ratio and successfully formed uniform 3D spheroids in ultra-low-attachment black 96-well plates with flat, clear bottoms after 2 d. Flow cytometry analysis (Fig. S17) confirmed the presence of both 4T1 and 3T3 cells within the 3D spheroids. Following treatment of the spheroids with different formulations for 24 h, their morphology was observed by confocal microscopy under bright-field mode (Fig. S18). Notably, spheroids treated with TM@cP/siF-ErN showed a tendency to disintegrate from the periphery, indicating structural disruption induced by the treatment. This effect was even more pronounced in the group treated with TM@cP/siF-ErN following pre-irradiation with an 808 nm laser (TM@cP/siF-ErN + L), where spheroids gradually decreased in size and exhibited lower cell density compared with the non-irradiated group. The collagen I concentration in the 3D spheroids was then measured (Fig. 3E). Compared with the control, the TM@cP/siF-N + L group showed no significant change in collagen I levels, whereas TM@cP/siF-ErN + L treatment markedly reduced collagen I content (*P* < 0.001), demonstrating that ErN was successfully released, targeted 3T3 cells, and suppressed collagen I secretion. Moreover, apoptosis of 4T1 cells within the 3D spheroids was evaluated by FCM (Fig. 3F). The results demonstrated that, compared with the TM@cP/siF-N + L group, 4T1 cells within spheroids treated with TM@cP/siF-ErN + L exhibited a significantly higher apoptosis rate, approaching that observed in the cP/siF + ErN group. This effect is attributed to the ErN-mediated reduction of collagen I. Moreover, the apoptosis rate in the TM@cP/siF-ErN + L group was higher than in the TM@cP/siN-ErN + L group, confirming that cP/siF is critical for reversing anoikis resistance. The 3D model thus demonstrated that nano-cracker-induced anoikis in 4T1 cells results from the synergistic interaction between 4T1 tumor cells and 3T3 fibroblasts, both of which are essential.

### 3.4 In vivo accumulation and heterologous targeting delivery of TM@cP/siF-ErN driven by photothermal effect

**Fig. 4:**
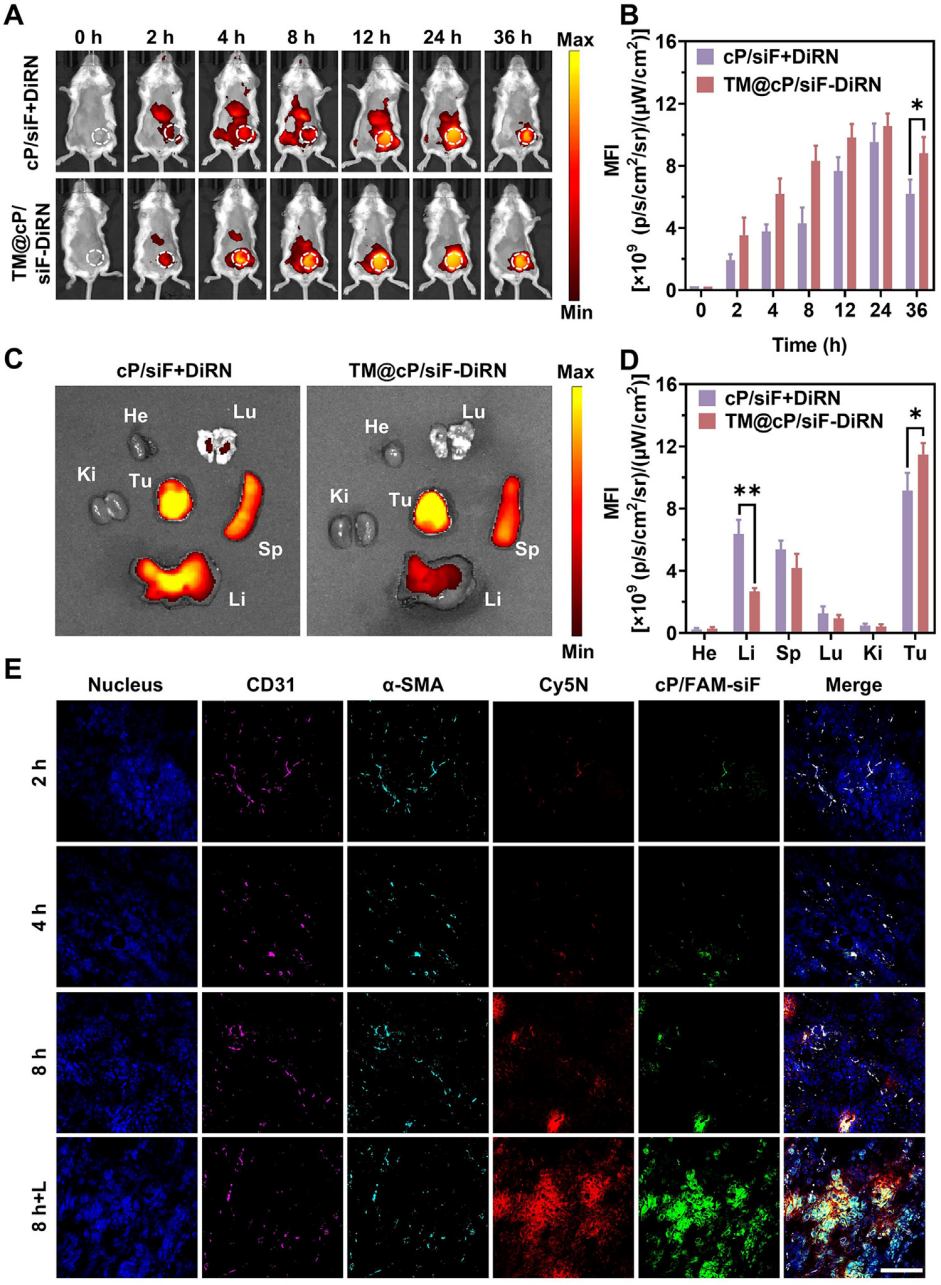
*In vivo* biodistribution and heterologous targeting ability of nano-cracker. (A) *In vivo* fluorescence images of 4T1 tumor-bearing BALB/c mice at different time points after intravenous injection of the mixture of cP/siF and ErN (cP/siF + ErN) or TM@cP/siF-ErN and (B) corresponding quantification (*n* = 3); (C) *Ex vivo* fluorescence imaging of tumors and major organs excised from the mice at 36 h post injection and (D) corresponding quantification (*n* = 3); (E) CD31 and α-SMA immunofluorescence staining of tumor sections after treated with nano-crackers at 2 h, 4 h and 8 h with or without irradiation post-injection. cP/siF and nanodisc were labeled by FAM (green) and Cy5 (red) separately. Blue: DAPI stained cell nuclei. Pink: CD31 stained by Alexa Fluor® 594-anti-CD31. Sky-blue: α-SMA stained by iF546-anti-α-SMA. Scale bar: 100 µm. Data are presented as mean ± SD.

To investigate the biodistribution of TM@cP/siF-ErN, 4T1 tumor-bearing mice were intravenously injected with DiR-labeled nano-crackers (TM@cP/siF-DiRN) and tracked using IVIS. As illustrated in Fig. 4A and 4B, both the mixture of cP/siF and DiRN (cP/siF + DiRN) and TM@cP/siF-DiRN displayed evident tumor accumulation. The accumulation of cP/siF + DiRN was attributed to the passive targeting of nanosized particles, while TM@cP/siF-DiRN showed significantly higher tumor accumulation at 36 h compared with the mixture group (*P* = 0.0308). Moreover, the mean fluorescence intensity of TM@cP/siF-DiRN showed essentially no further increase after 8 h, indicating this as the optimal time point for subsequent laser irradiation. At 36 h post-administration, the major organs were collected for *ex vivo* fluorescence imaging. In the cP/siF + DiRN group, fluorescence was mainly observed in the liver and spleen, in addition to the tumor, likely due to the small particle size of cP/siF and DiRN (Fig. 4C). In contrast, TM@cP/siF-DiRN showed reduced accumulation in the liver while significantly increasing tumor accumulation compared with cP/siF + DiRN (Fig. 4D). These results demonstrate that M1M coating enhances tumor-specific drug distribution and minimizes off-target accumulation in other organs.

After accumulating in tumor tissue, the heterologous targeting ability of the nano-crackers was evaluated by immunofluorescence staining of tumor tissues from 4T1 tumor-bearing mice at 2 h, 4 h and 8 h with or without laser irradiation post-injection. As illustrated in Fig. 4E, cP/FAM-siF and Cy5N were labeled with FAM and Cy5, respectively, while blood vessels were stained with Alexa Fluor® 594-conjugated anti-CD31 and iF546-conjugated anti-α-SMA antibodies. Limited fluorescence of cP/FAM-siF and Cy5N was observed at 2 h and 4 h. Without laser irradiation at 8 h, the fluorescence of cP/FAM-siF and Cy5N was co-localized with blood vessels and mainly distributed around them. Upon mild-temperature laser irradiation, the fluorescence began to separate and disperse throughout the tumor tissue. These results indicate that laser irradiation not only triggered the detonation of the nano-crackers to achieve heterologous targeting delivery but also promoted their extravasation from blood vessels. This effect is attributed to nanoparticle accumulation and the formation of a blood pool. Previous studies have shown that the dense basement membrane surrounding tumor vasculature severely limits nanomedicine extravasation, causing particles to accumulate near vessels and form blood pool-like structures [35]. Treatment with 808 nm laser irradiation facilitated the rupture of the nano-crackers, promoting their extravasation from the blood pool into the inner tumor tissue. Co-localization analysis between cP/FAM-siF and tumor cells was then performed. In the group treated with TM@cP/FAM-siF-Cy5N for 8 h without laser irradiation, the Pearson’s coefficient was only 0.21. After laser exposure, Pearson’s coefficient increased to 0.60, indicating strong co-localization and demonstrating that cP/FAM-siF effectively targeted tumor cells.

### 3.5 In vivo antitumor efficacy

**Fig. 5:**
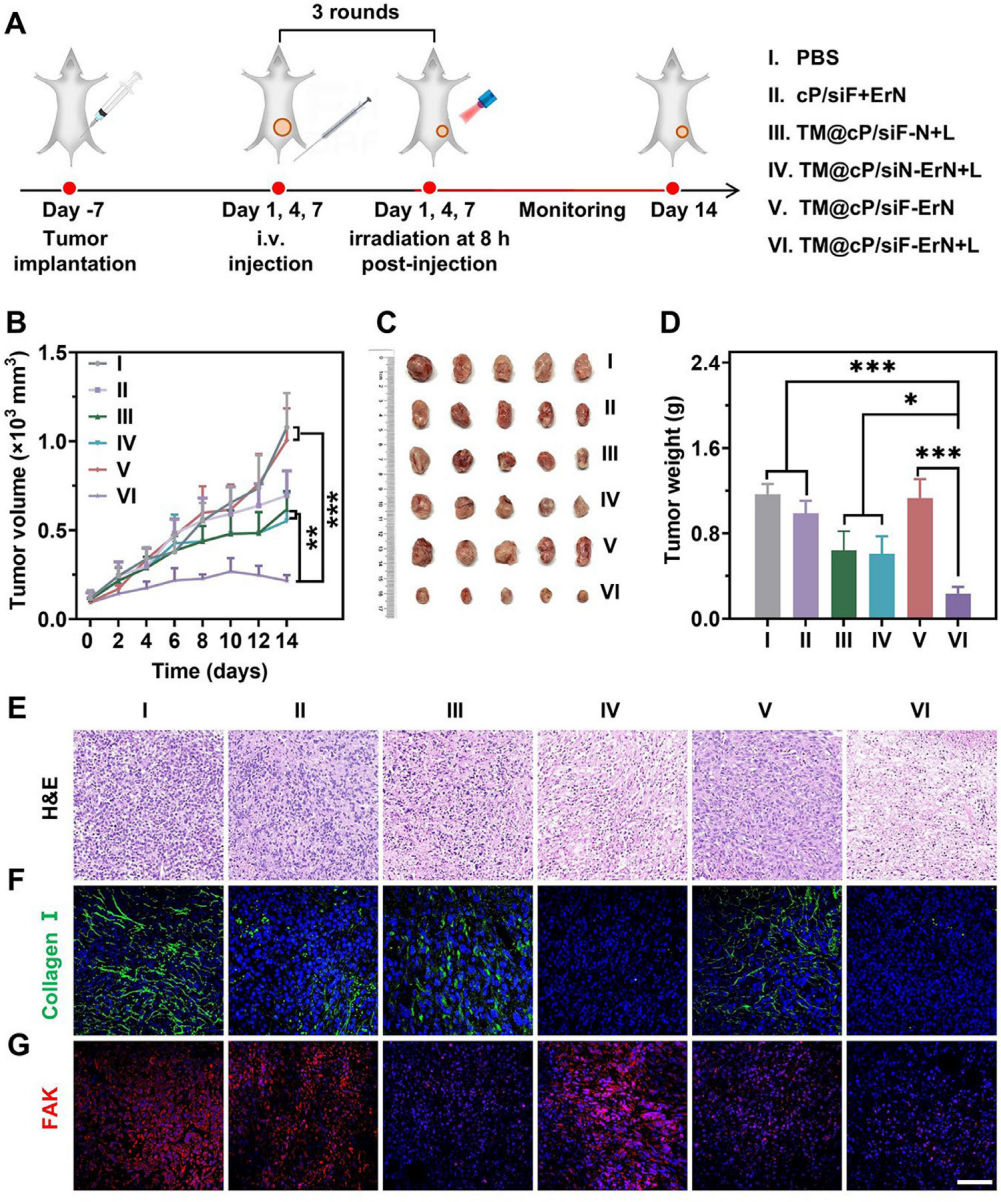
*In vivo* antitumor effect of nano-crackers on 4T1 tumor-bearing mice. (A) Therapeutic schedule and groups for antitumor efficacy study; (B) Tumor growth curves and (C) photograph of tumor tissues at the end of treatments of the 4T1 tumor-bearing mice after different treatments (*n* = 5); (D) Tumor weights of different groups (*n* = 5); (E) H&E staining of tumor tissues after different treatments; (F) Collagen I and (G) FAK immunofluorescence staining of tumor sections after treatments in different groups. Scale bar: 100 µm. Data are presented as mean ± SD.

Given the excellent biodistribution and tumor accumulation of the nano-crackers, their *in vivo* antitumor efficacy was further evaluated in unilateral 4T1-bearing female BALB/c mice. Mice were randomly assigned to six groups: PBS, cP/siF + ErN, TM@cP/siF-N + L, TM@cP/siN-ErN + L, TM@cP/siF-ErN, and TM@cP/siF-ErN + L. The treatment protocol is illustrated in Fig. 5A. As illustrated in Fig. 5B–5D, treatment with cP/siF + ErN exhibited limited antitumor activity, likely due to the asynchronous effects of siFAK and erianin, which have distinct pharmacokinetic profiles. Both TM@cP/siF-N + L and TM@cP/siN-ErN + L, containing a single active agent, demonstrated moderate antitumor effects, attributable to the action of siFAK or ErN on tumor cells or CAFs, respectively, combined with the repolarization effect of M1M debris. Without laser irradiation, TM@cP/siF-ErN showed almost no inhibition of tumor progression, as most of the nano-crackers were internalized by tumor cells, and the effectiveness of siFAK was restricted in adherent tumor cells. Only upon laser irradiation did TM@cP/siF-ErN demonstrate superior therapeutic efficacy, owing to the synergistic effects of Er and siFAK. To further evaluate this synergy, tumor tissues were subjected to H&E staining. As illustrated in Fig. 5E, the tumor tissue from the TM@cP/siF-ErN + L group exhibited pronounced cellular damage, including nuclear shrinkage. In addition, the inhibition of collagen I synthesis and FAK knockdown efficiency *in vivo* were assessed via immunofluorescence staining of the tumor tissues. Consistent with the *in vitro* findings, the green fluorescence in erianin-containing groups decreased, indicating effective inhibition of collagen I synthesis (Fig. 5F). Similarly, the red fluorescence in siFAK-containing groups was reduced, reflecting efficient FAK knockdown (Fig. 5G). The preliminary safety of TM@cP/siF-ErN in tumor-bearing mice was also evaluated. No significant changes in body weight were observed, indicating good systemic tolerance (Fig. S19), and H&E staining of major organs revealed no notable tissue abnormalities after treatment (Fig. S20). These results collectively demonstrate that the nano-cracker-mediated “wandering tumor cells” strategy is both effective and safe *in vivo* in 4T1 tumor-bearing mice.

**Fig. 6:**
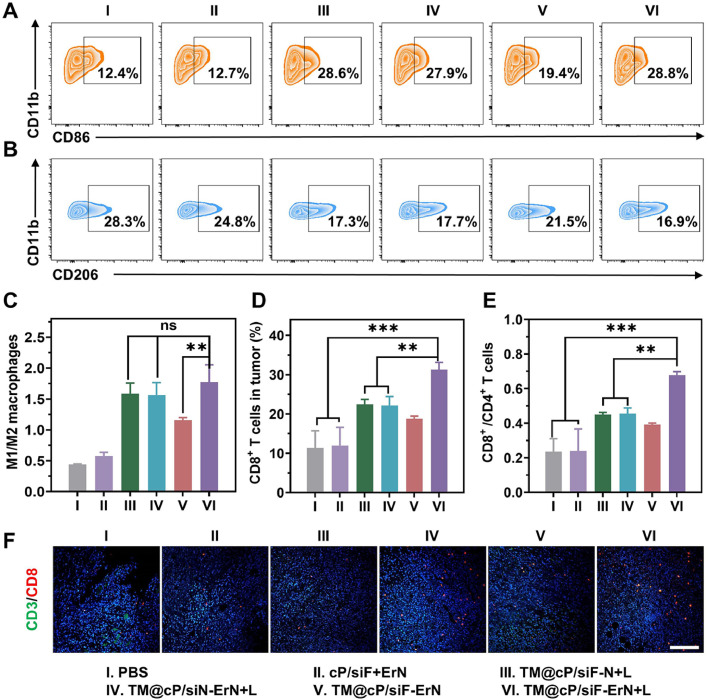
*In vivo* macrophage repolarization and immune infiltration effect. (A, B) Representative flow cytometric assay of M1 (A) and M2 (B) macrophages in tumor tissues induced by different treatments; (C) The ratio of M1 and M2 macrophages in tumor tissues quantitatively analyzed by FCM (*n* = 3); (D) Corresponding quantitative analysis of intratumoral infiltration of CD8^+^ T cells and (E) the ratio of CD8^+^ and CD4^+^ T cells in tumor tissues (*n* = 3); (F) CD3^+^CD8^+^ T cells immunofluorescence staining of tumor sections after different treatments. Scale bar: 100 µm. Data are presented as mean ± SD.

Furthermore, the *in vivo* experiments confirmed that laser-induced M1M debris could amplify the polarization effect of M1M. The repolarization of macrophages within tumor tissues was further assessed by FCM (Fig. S21). As illustrated in Fig. 6A and 6B, all groups treated with TM exhibited macrophage repolarization, characterized by an increased proportion of M1-type macrophages and a decreased proportion of M2-type macrophages. Notably, the repolarization effect of the TM@cP/siF-ErN group was further enhanced following laser irradiation, owing to the generation of additional M1M debris (Fig. S22), demonstrating that laser treatment can significantly amplify the macrophage polarization effect. Furthermore, the ratio of M1 to M2 macrophages in tumor tissues indicated that TM@cP/siF-ErN + L treatment increased the proportion of M1-type macrophages, thereby promoting antitumor immune responses (Fig. 6C). The secretion of cytokines from macrophages was also evaluated, showing increased levels of M1 markers (TNF-α and iNOS) and reduced levels of M2 markers (TGF-β1 and Arg1) (Fig. S23). In addition, the reduction of collagen I facilitated the infiltration of immune cells [24]. The proportions of CD4^+^ and CD8^+^ T cells within tumor tissues were further quantified by FCM (Fig. S24). As illustrated in Figs. 6D and S25, the TM-containing groups exhibited a higher proportion of CD8^+^ T cells in tumor tissues. The ratio of CD8^+^ to CD4^+^ T cells was significantly increased in the TM@cP/siF-ErN + L group compared with other groups, indicating that this treatment could effectively activate a CD8^+^ T cell-mediated antitumor immune response (Fig. 6E). Additionally, the increased presence and distribution of CD3^+^CD8^+^ T cells were confirmed in immunofluorescence-stained tumor sections of the TM@cP/siF-ErN + L group (Fig. 6F). These results demonstrate that the nano-cracker not only enables heterologous targeting but also effectively stimulates the immune system, thereby enhancing overall antitumor efficacy.

## 4 Conclusion

In summary, we proposed a “wandering tumor cells” strategy to remodel the tumor ECM by targeting CAFs and inducing tumor cell anoikis for enhanced antitumor therapy. To implement this strategy, we developed a photothermally detonated, macrophage membrane-camouflaged nano-cracker for heterologous delivery of erianin and siFAK. Both *in vitro* and *in vivo* experiments demonstrated that the nano-cracker effectively targeted tumor tissues and, upon laser irradiation, was detonated into ErN, cP/siF and M1M debris. This triggered inhibition of collagen I synthesis in CAFs and reversal of anoikis resistance in tumor cells, achieving synergistic antitumor effects. Additionally, the M1M debris generated by laser irradiation amplified the repolarization of M2-type macrophages, which, together with T-lymphocyte infiltration triggered by collagen I downregulation, enhanced the antitumor immune response. The nano-cracker not only achieved superior tumor inhibition but also demonstrated potential for heterologous targeting drug delivery.

## Data availability

Data will be made available on request.

## CRediT authorship contribution statement

**Shengjie Sun:** Writing – original draft, Validation, Methodology, Investigation, Data curation, Conceptualization. **Simin Wen:** Writing – original draft, Validation, Methodology, Investigation, Formal analysis, Data curation. **Ruiqi Zhang:** Validation, Methodology, Investigation. **Yanan Fu:** Validation, Methodology, Investigation. **Huisong Hao:** Validation, Methodology, Investigation. **You Li:** Validation, Methodology, Investigation. **Yingfei Wen:** Validation, Methodology, Investigation. **Ying Huo:** Validation, Methodology, Investigation. **Yixuan Fang:** Validation, Supervision, Investigation. **Shihao Zhuang:** Validation, Supervision, Investigation. **Jia Tang:** Validation, Supervision, Investigation. **Yanglong Hou:** Supervision, Resources, Investigation. **Tianqi Wang:** Writing – review & editing, Supervision, Resources, Funding acquisition, Conceptualization. **Meiying Wu:** Writing – review & editing, Supervision, Resources, Project administration, Funding acquisition.

## Conflicts of interest

The authors declare that there is no conflicts of interest.

## Appendix Supplementary materials

Image, application 1
