# Supplementary material for: Photothermal-detonated functional macrophage membrane-camouflaged nano-crackers induce tumor cell wandering-anoikis
Source: Asian J Pharm Sci. 2026 Jun 4;21(3):101171. doi: 10.1016/j.ajps.2026.101171 (PMC13284475; doi:10.1016/j.ajps.2026.101171)
Supplement: Supplementary file 1 [file mmc1.docx]

Supplementary Material for

**Photothermal-Detonated Functional Macrophage Membrane-Camouflaged Nano-Crackers Induce Tumor Cell Wandering-Anoikis**

Shengjie Sun^a,1^, Simin Wen^a,1^, Ruiqi Zhang^a^, Yanan Fu^a^, Huisong Hao^a^, You Li^c^, Yingfei Wen^c^, Ying Huo^a^, Yixuan Fang^a^, Shihao Zhuang^a^, Jia Tang^a^, Yanglong Hou^b,*^, Tianqi Wang^a,*^, Meiying Wu^a,*^

^a^School of Pharmaceutical Sciences (Shenzhen), Shenzhen Campus of Sun Yat-sen University, Shenzhen 518107, China

^b^School of Materials, Shenzhen Campus of Sun Yat-sen University, Shenzhen 518107, China

^c^The Seventh Affiliated Hospital, Sun Yat-sen University, Shenzhen 518107, China

^*^Corresponding authors.

E-mail addresses: hou@mail.sysu.edu.cn; wangtq6@mail.sysu.edu.cn; [wumy53@mail.sysu.edu.cn](mailto:wumy53@mail.sysu.edu.cn)

^1^These authors contributed equally to this work.

**Supplementary Figures**


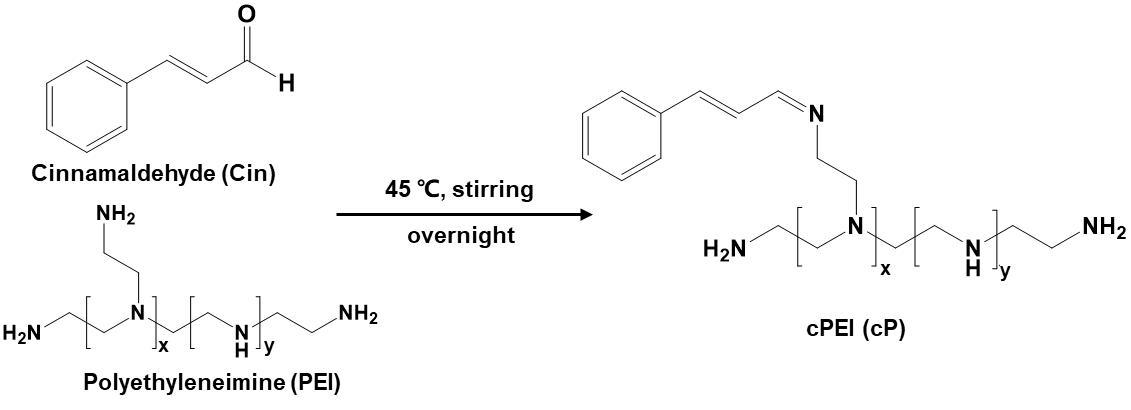


**Fig. S1.** The synthesis procedure of cP.


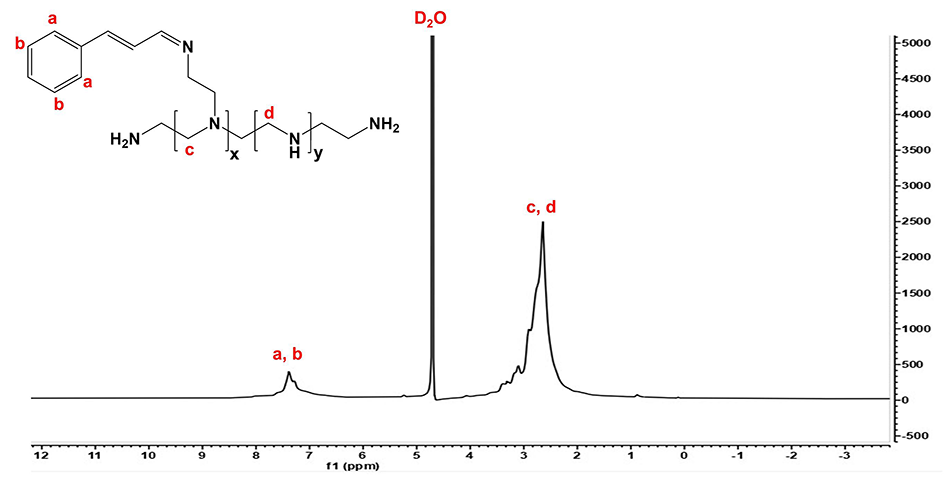


**Fig. S2.** ^1^H NMR spectrum of cP.


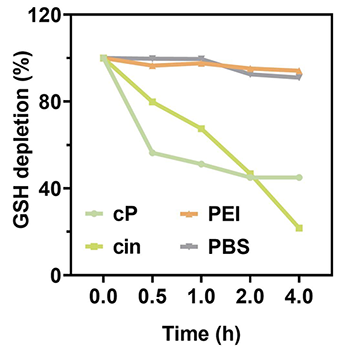


**Fig. S3.** The GSH depletion ability of PBS, cin, PEI and synthesized cP.


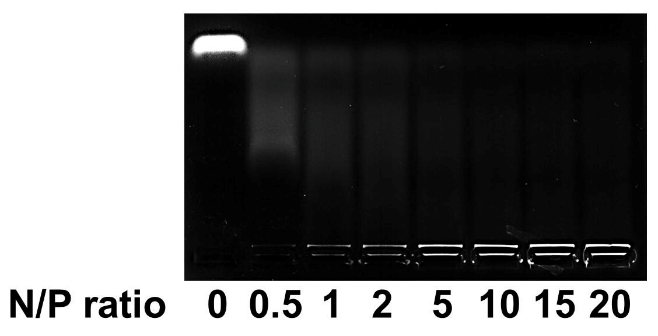


**Fig. S4.** Agarose gel electrophoresis of free siFAK before encapsulation and cP/siF complex with different N/P ratios.


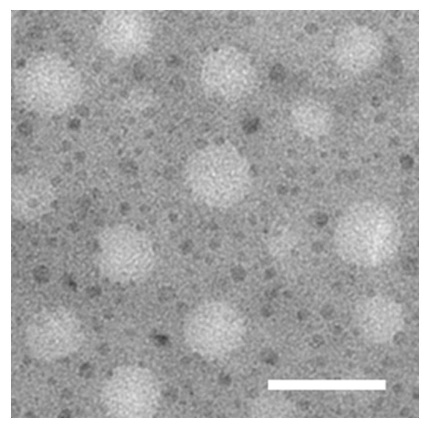


**Fig. S5.** TEM image of cP/siF. Scale bar, 100 nm.


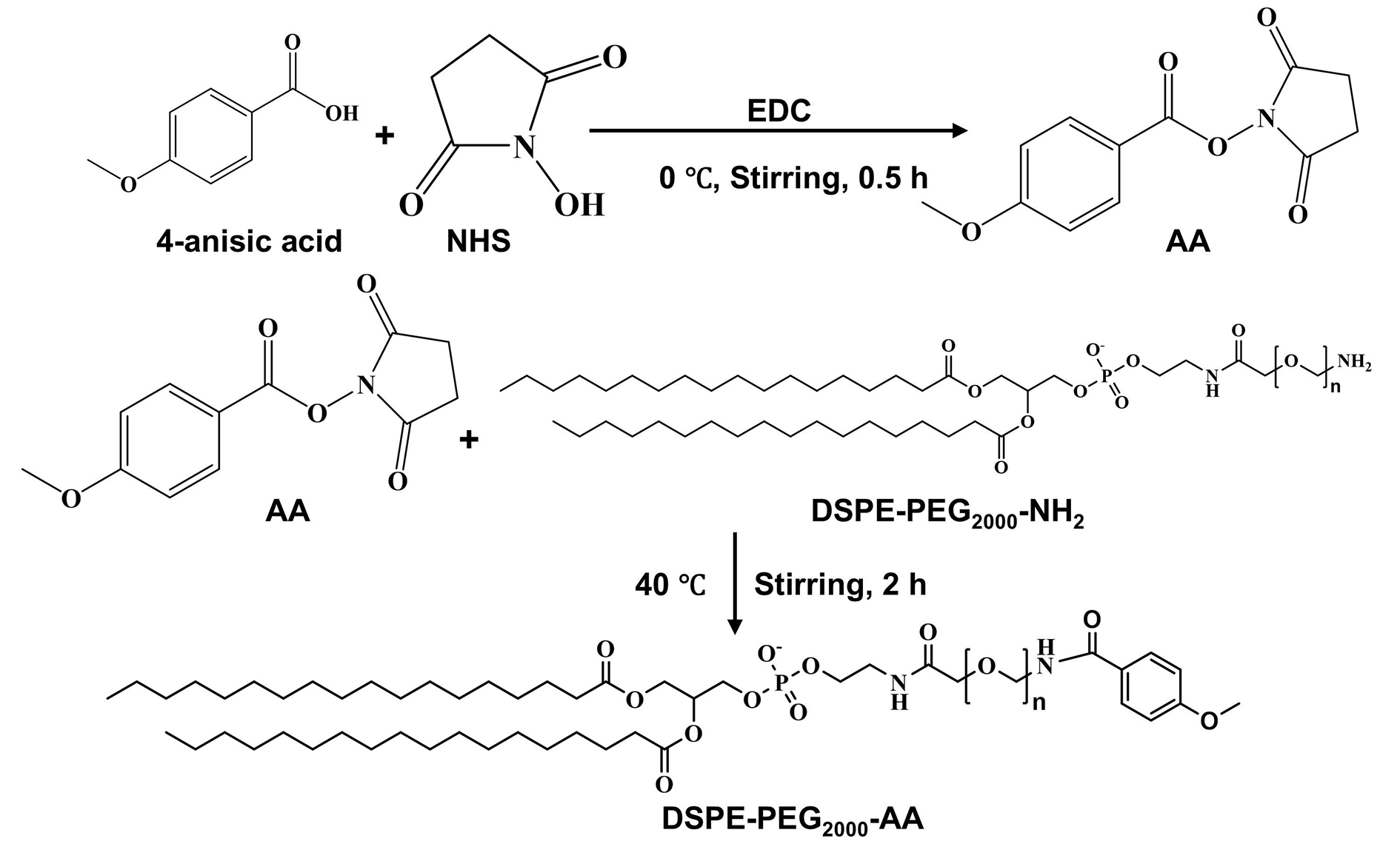


**Fig. S6.** The synthesis procedure of DSPE-PEG_2000_-AA.


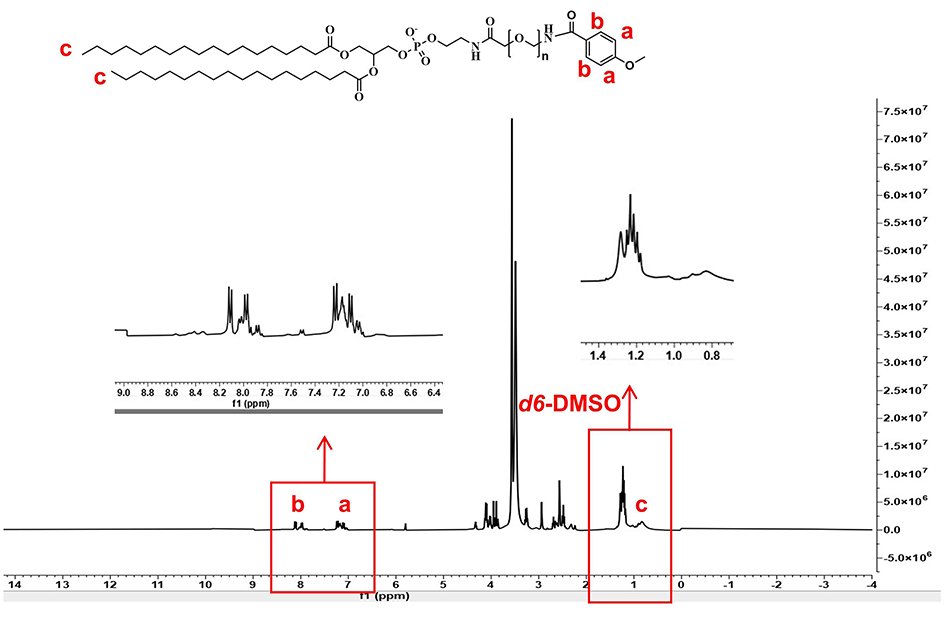


**Fig. S7.** ^1^H NMR spectrum of DSPE-PEG_2000_-AA.


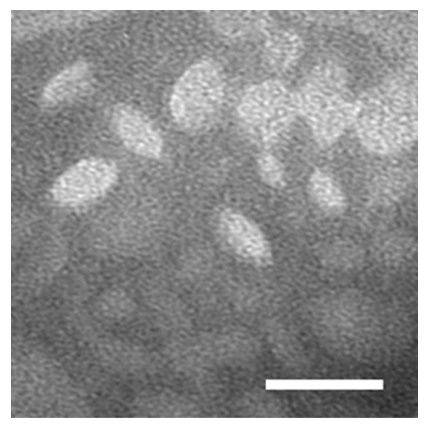


**Fig. S8.** TEM image of ErN. Scale bar, 50 nm.


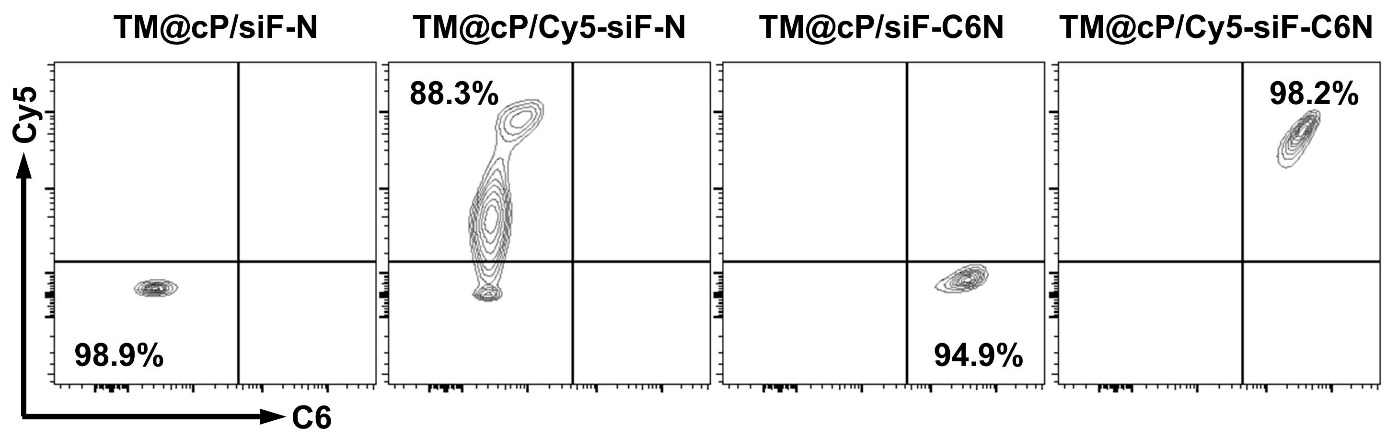


**Fig. S9.** Colocalization analysis of fluorescently labeled siF (Cy5-siF) and nanodiscs (labeled with C6) in TM@cP/Cy5-siF-C6N.


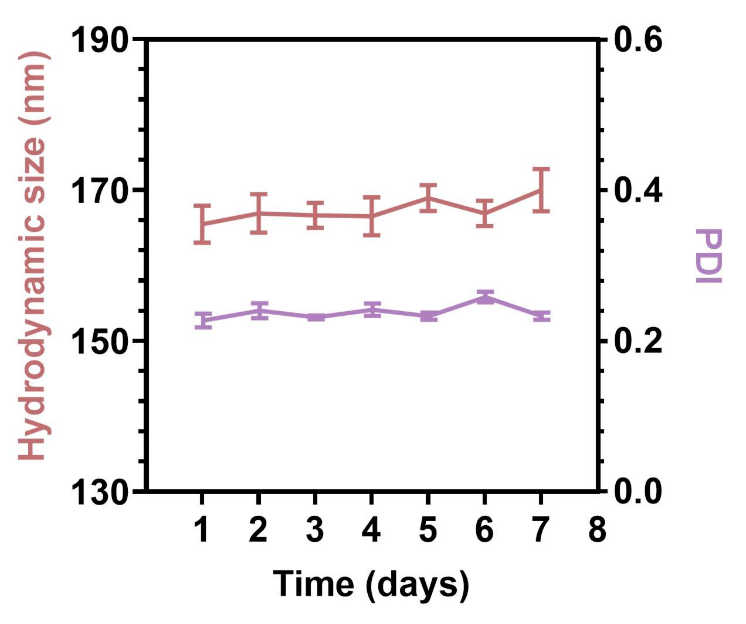


**Fig. S10.** Stability of TM@cP/siF-ErN in PBS for 7 days. Data are presented as mean ± SD. (*n* = 3)


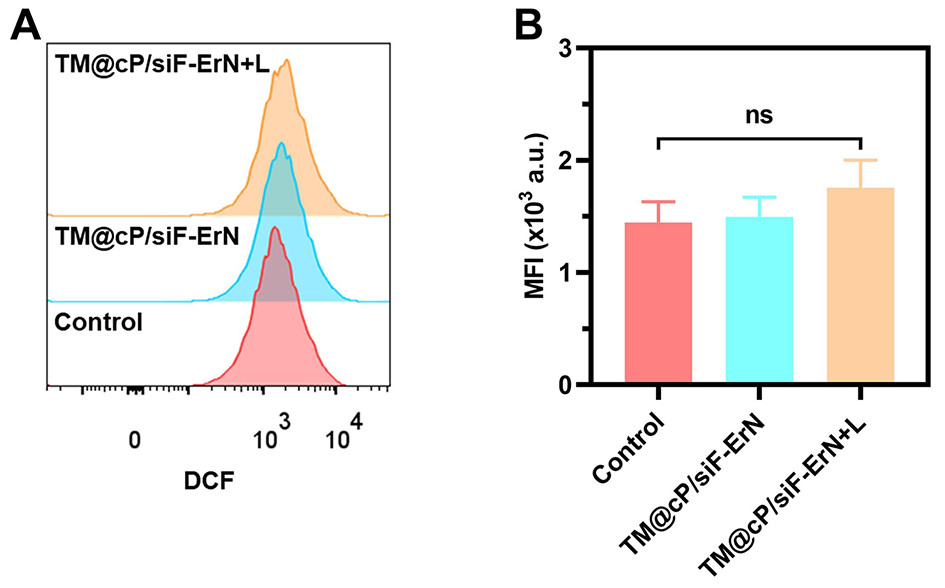


**Fig. S11.** Flow cytometry analysis (A) of intracellular ROS generation and the quantitative analysis (B). Data are presented as mean ± SD (*n* = 3).


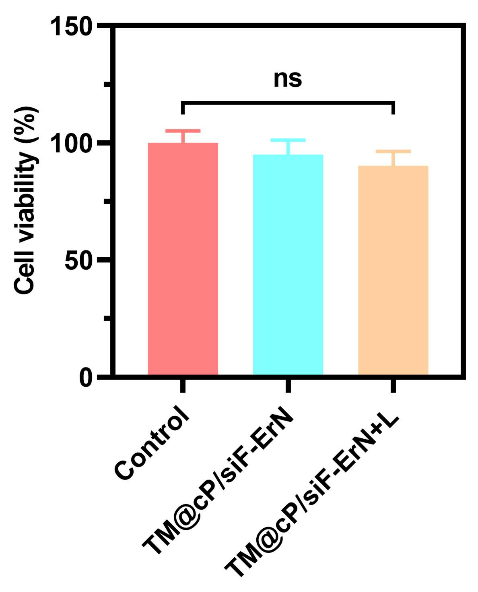


**Fig. S12.** Cytotoxicity of 4T1 cells with different treatments for 8 h. Data are presented as mean ± SD (*n* = 5).


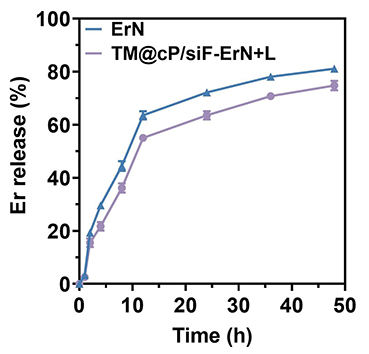


**Fig. S13.** The Er release profiles of the different formulations. Data are presented as mean ± SD. (*n* = 3)


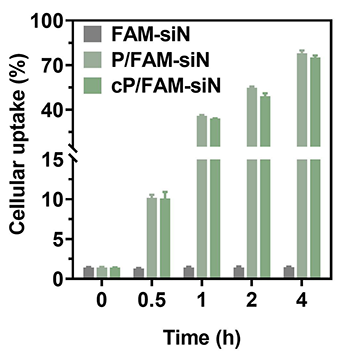


**Fig. S14.** Flow cytometric analysis of cellular uptake of cP/FAM-siN in 4T1 cells at different time points. Data are presented as mean ± SD. (*n* = 3)


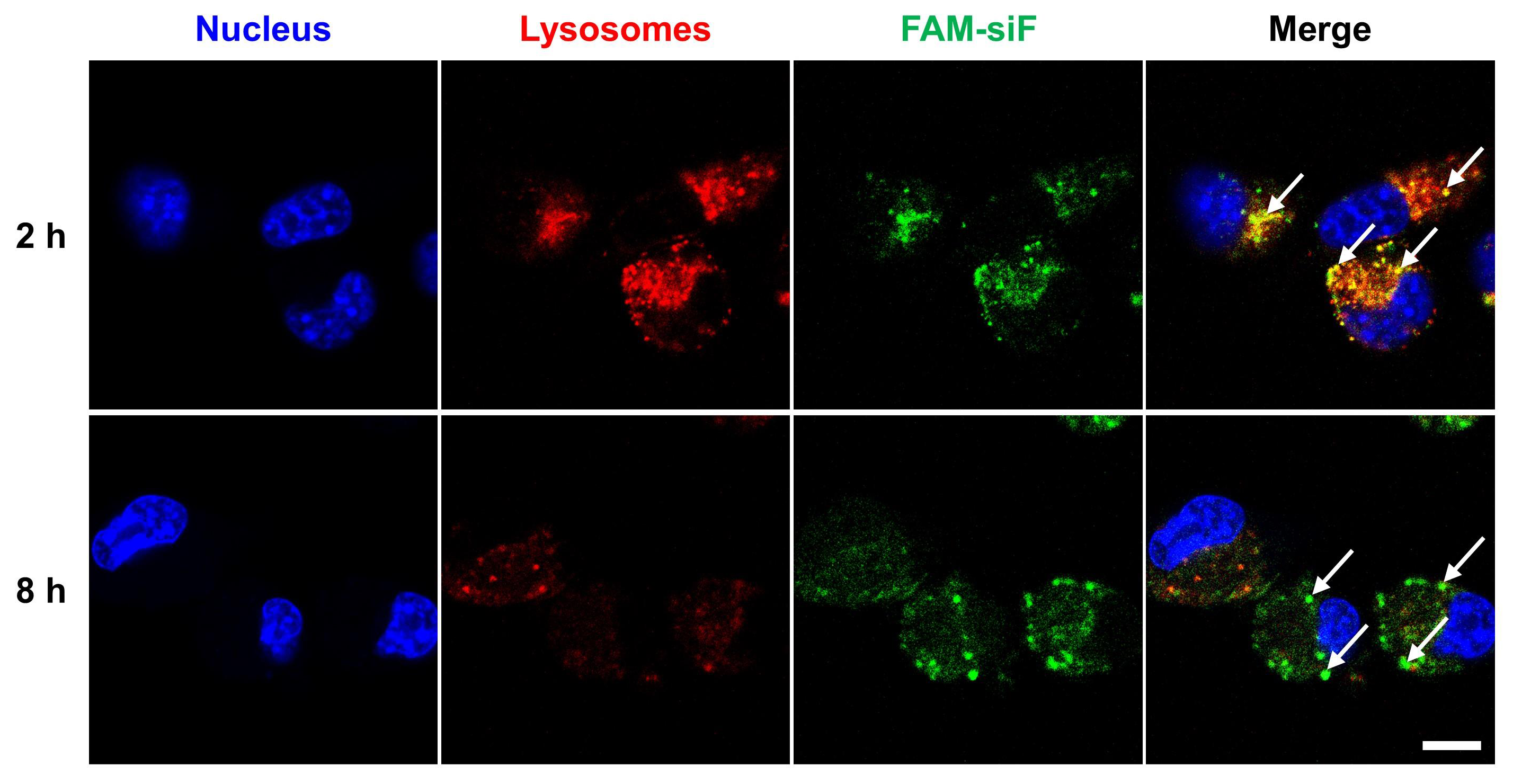


**Fig. S15.** The localization analysis of lysosome (red) and cP/FAM-siF (green) in 4T1 cells at 2 and 8 h after administration. Blue: Hoechst 33342 stained cell nuclei. White arrows: colocalization of cP/FAM-siF and lysosomes at 2 h or the lysosomal escape at 8 h. Scale bar, 20 μm.


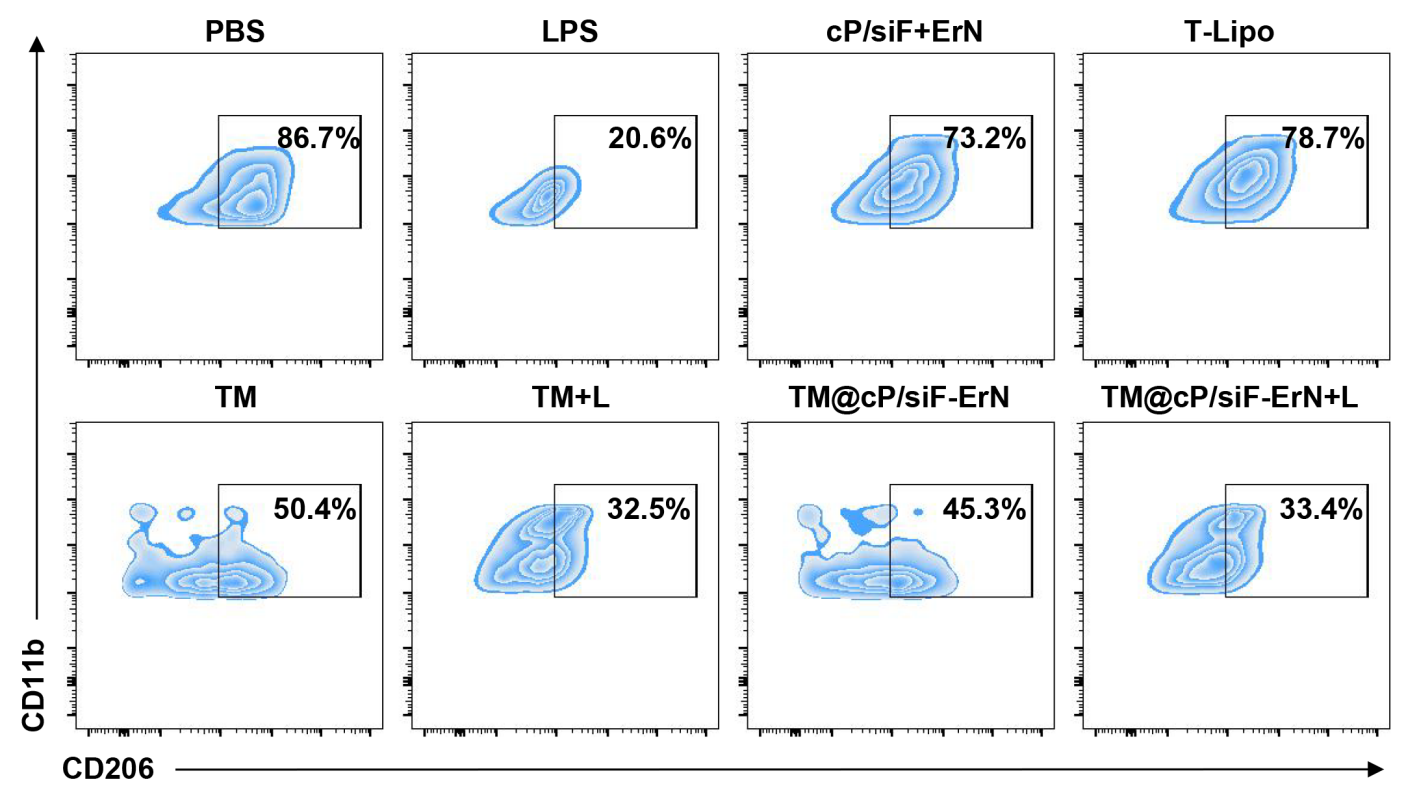


**Fig. S16.** The flow cytometric examination of M2-type macrophages (CD206^+^, gated on CD11b^+^) with different treatments.


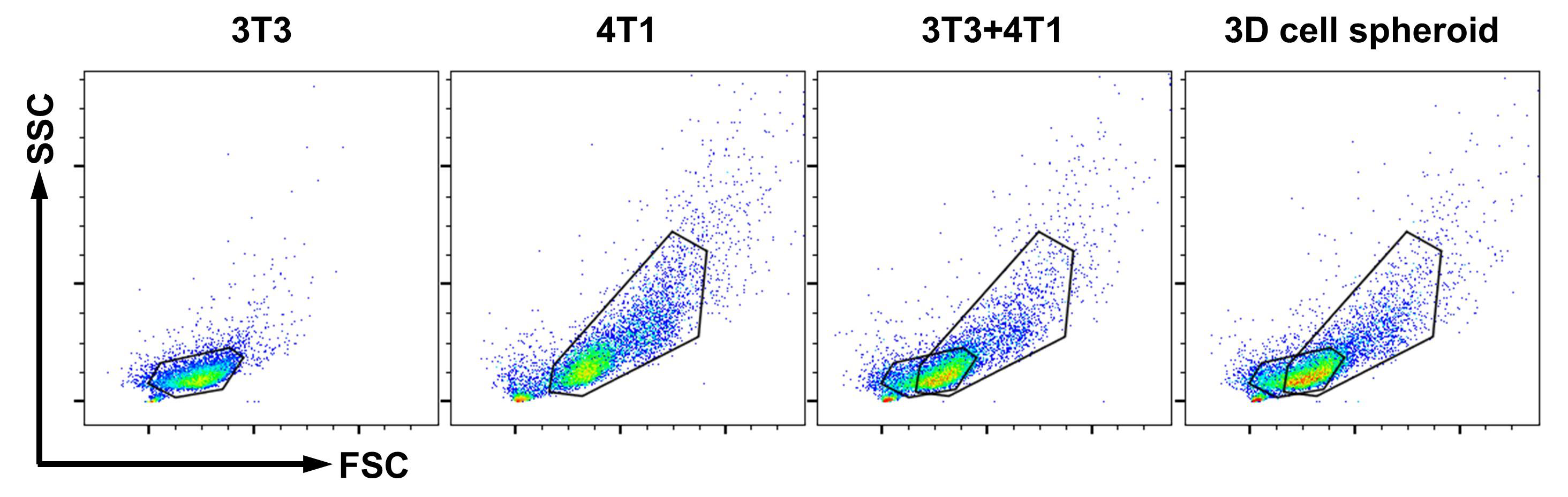


**Fig. S17.** The flow cytometry analysis of the formed 3D cell spheroid was performed with the following controls: suspensions of 3T3 cells alone, 4T1 cells alone, and a directly mixed suspension of both cell types.


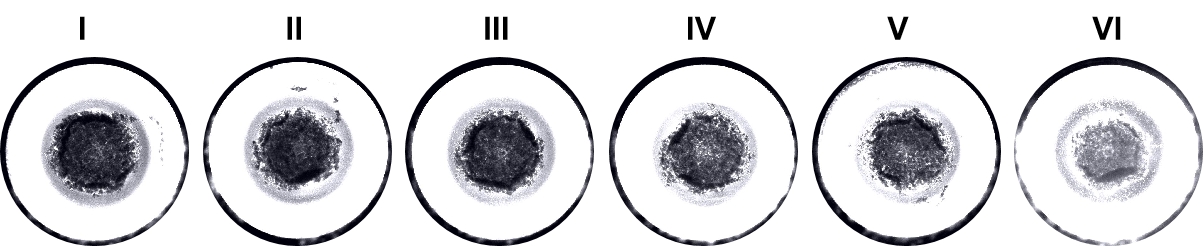


**Fig. S18.** The confocal images (10×) of 3D cell spheroid model. I. PBS, II. cP/siF+ErN, III. TM@cP/siF-N+L, IV. TM@cP/siN-ErN+L, V. TM@cP/siF-ErN, VI. TM@cP/siF-ErN+L.


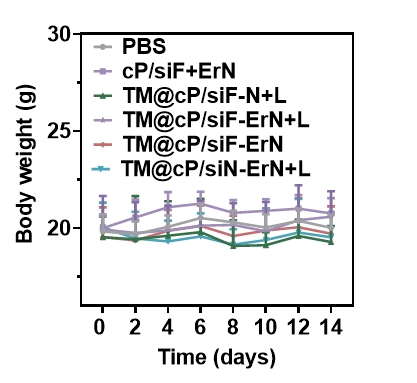


**Fig. S19.** The average body weights of mice after different treatments. Data are presented as mean ± SD (*n* = 5).


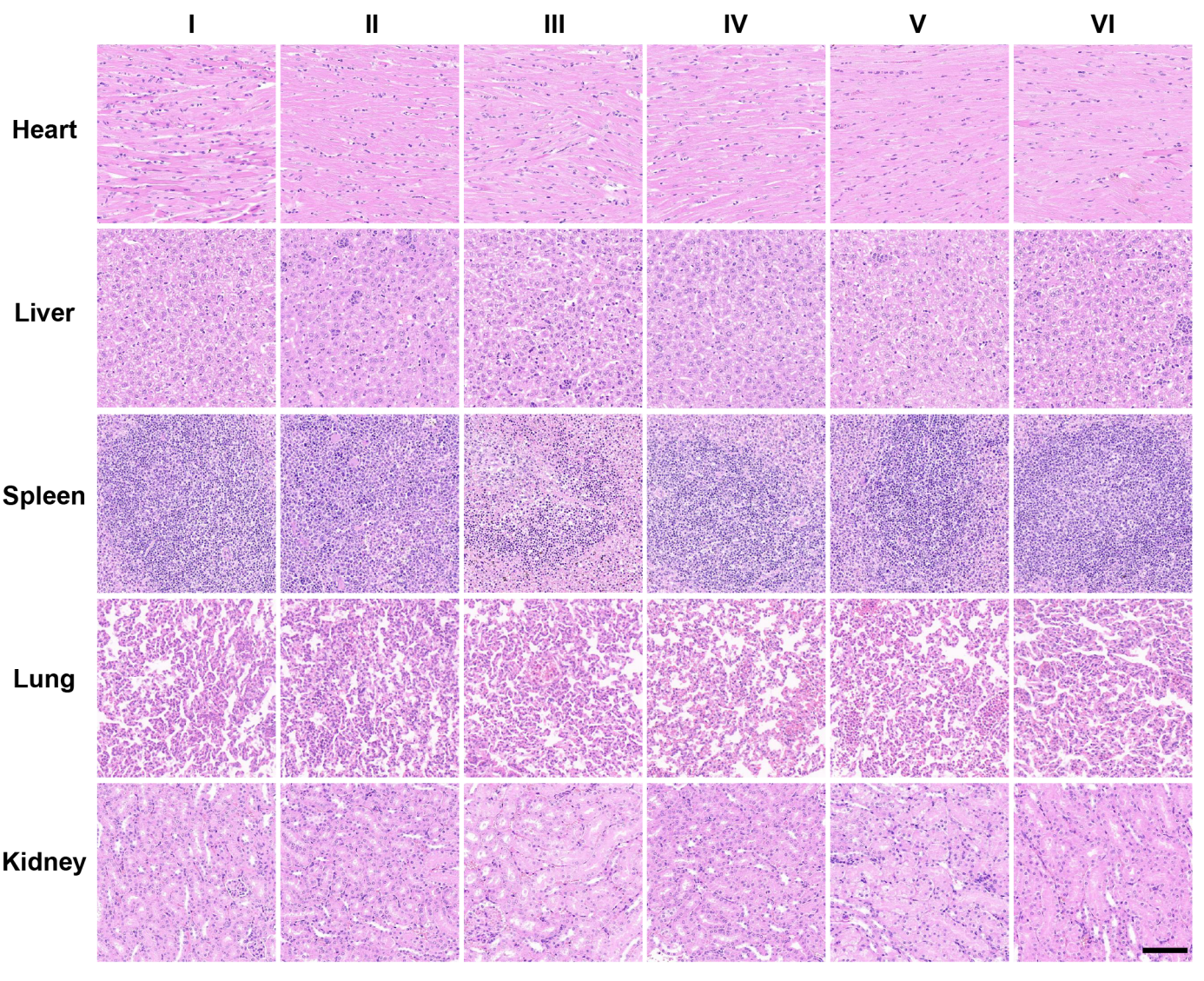


**Fig. S20.** H&E staining of main organs after different treatments. Scale bar, 100 μm. I. PBS, II. cP/siF+ErN, III. TM@cP/siF-N+L, IV. TM@cP/siN-ErN+L, V. TM@cP/siF-ErN, VI. TM@cP/siF-ErN+L.


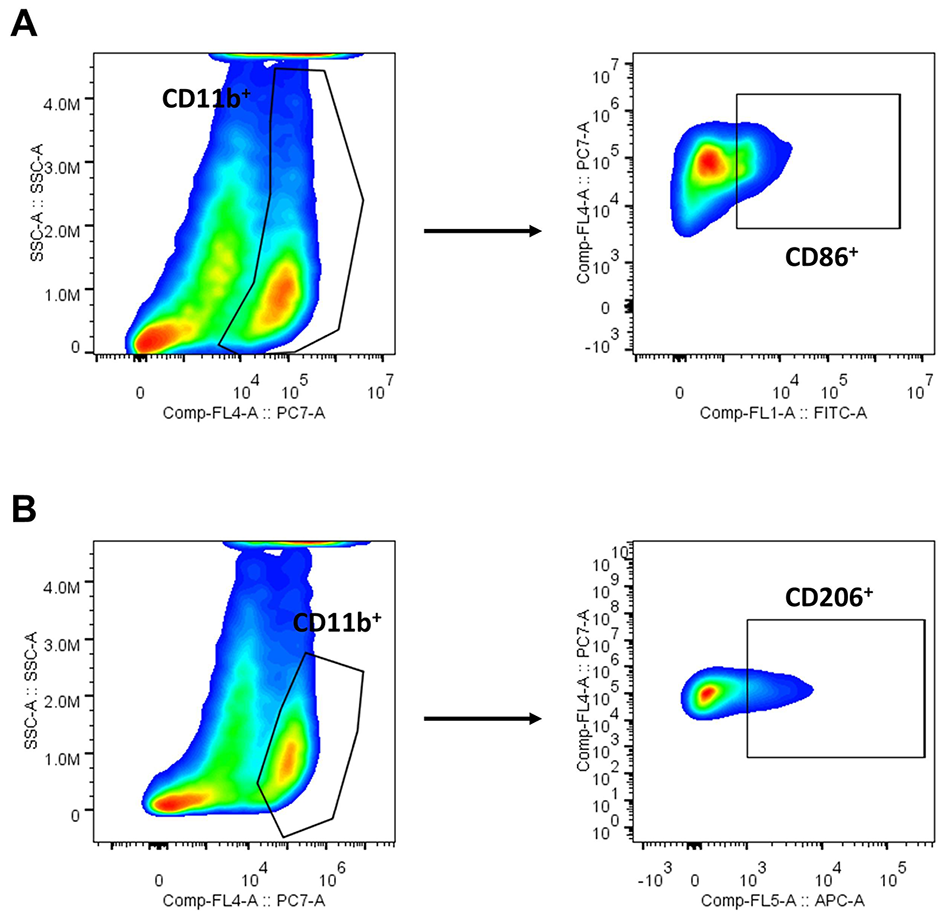


**Fig. S21.** Representative flow cytometry gating scheme for M1-type (A) and M2-type (B) macrophages in tumor tissue.


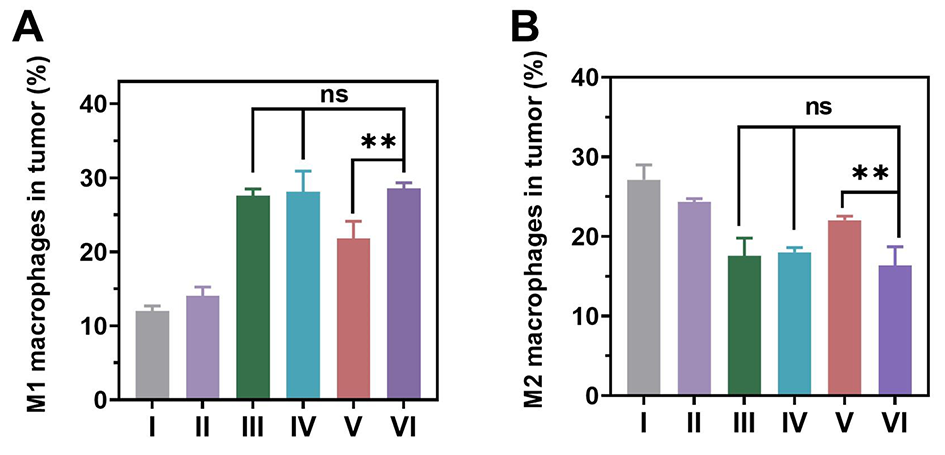


**Fig. S22.** Flow cytometric quantitative analysis of M1-type (A) and M2-type (B) macrophages in tumor tissue after different treatments. I. PBS, II. cP/siF+ErN, III. TM@cP/siF-N+L, IV. TM@cP/siN-ErN+L, V. TM@cP/siF-ErN, VI. TM@cP/siF-ErN+L. Data are presented as mean ± SD (*n* = 3).


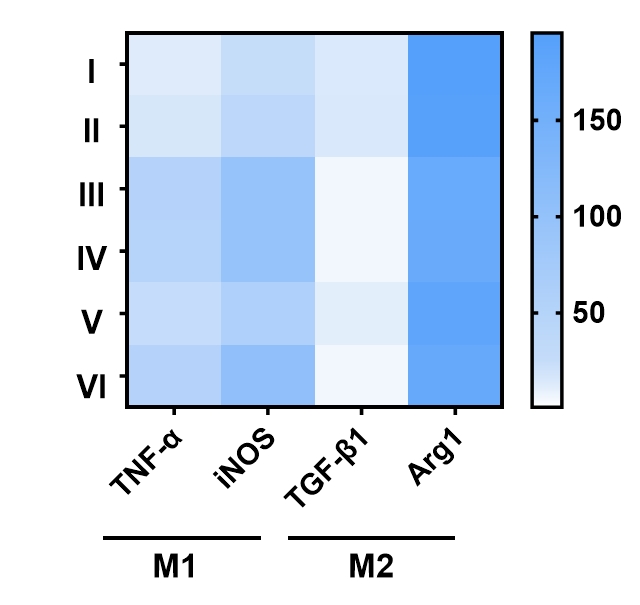


**Fig. S23.** Secretion of cytokines of M1-type and M2-type macrophages with different treatments of the tumors at the end of antitumor studies. Ⅰ. PBS, Ⅱ. cP/siF+ErN, Ⅲ. TM@cP/siF-N+L, Ⅳ. TM@cP/siN-ErN+L, Ⅴ. TM@cP/siF-ErN, Ⅵ. TM@cP/siF-ErN+L. (*n* = 3).


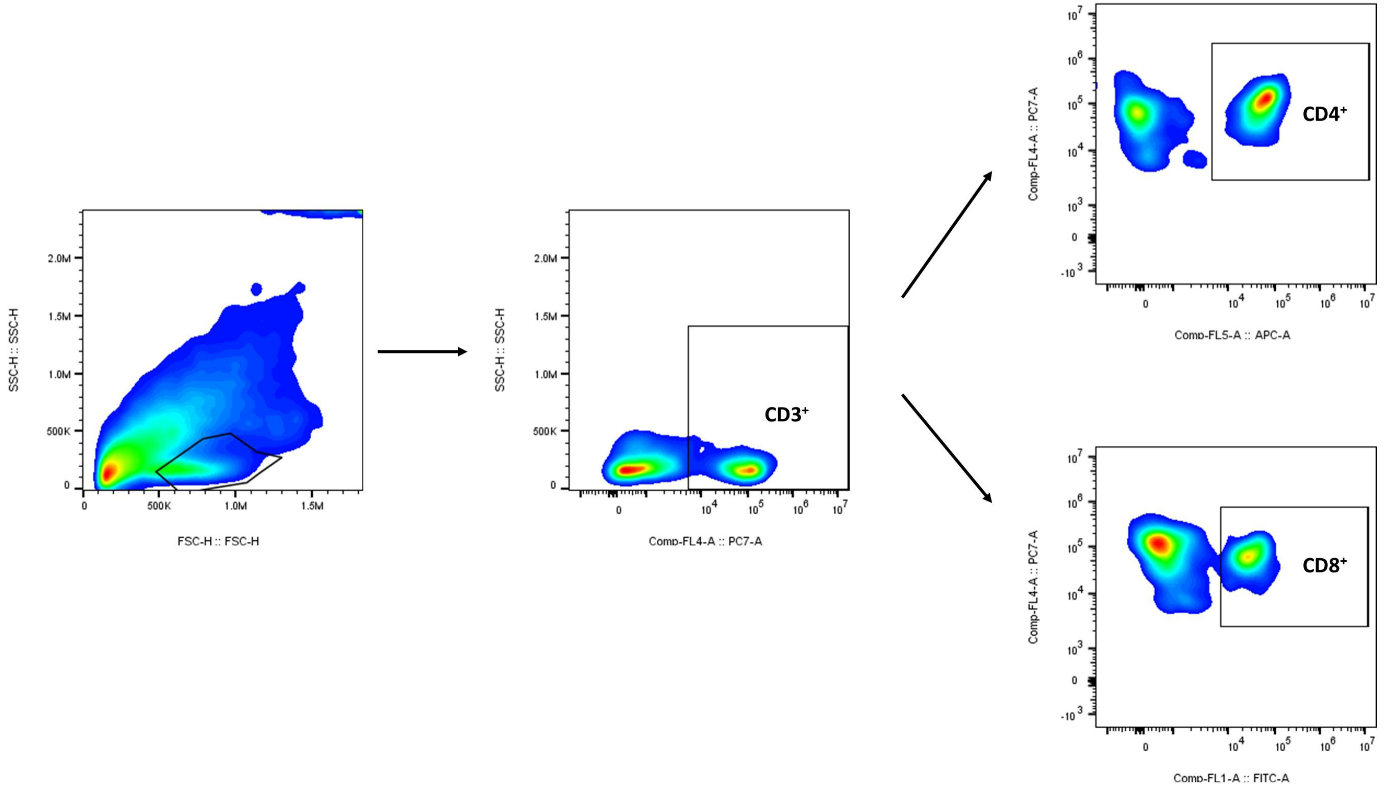


**Fig. S24.** Representative flow cytometry gating scheme for CD3^+^CD4^+^ and CD3^+^CD8^+^ T cells in tumor tissues.


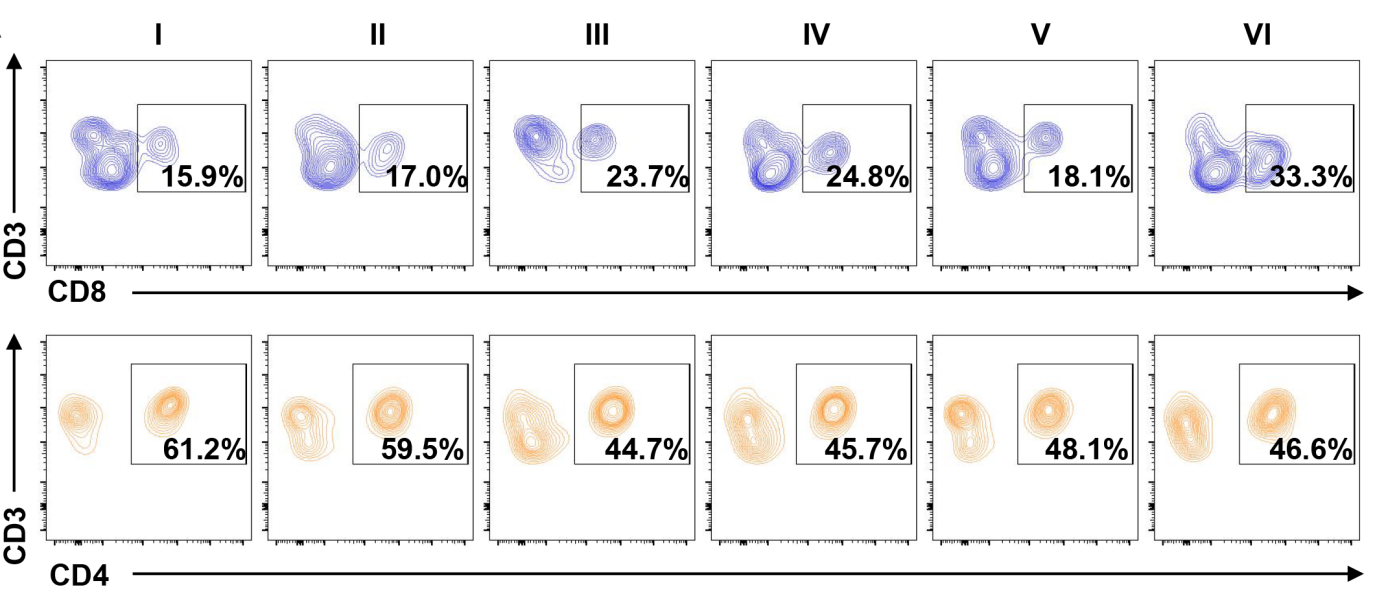


**Fig. S25.** Flow cytometric plots of the CD3^+^CD8^+^ and CD3^+^CD4^+^ T cells in tumor tissues at the end of treatment. Ⅰ. PBS, Ⅱ. cP/siF+ErN, Ⅲ. TM@cP/siF-N+L, Ⅳ. TM@cP/siN-ErN+L, Ⅴ. TM@cP/siF-ErN, Ⅵ. TM@cP/siF-ErN+L.

**Supplementary Table**

**Table S1.** Antibodies used for flow cytometry or immunofluorescence.

| Antibodies | Company | Clone No. |
| --- | --- | --- |
| Anti-CD16/32 | Biolegend | S17011E |
| Anti-CD11b (PE/Cyanine 7) | Biolegend | M1/70 |
| Anti-CD86 (FITC) | Biolegend | GL-1 |
| Anti-CD206 (APC) | Biolegend | C068C2 |
| Anti-CD31 (Alexa Fluor^®^ 594) | Biolegend | 390 |
| Anti-CD3 (PE/Cyanine 7) | Biolegend | 17A2 |
| Anti-CD4 (APC) | Biolegend | GK1.5 |
| Anti-CD8a (FITC) | Biolegend | 53-6.7 |
| Anti-FAK | Servicebio | GB11545 |
| Anti-α-SMA | Servicebio | / |
| Anti-Collagen I | Servicebio | GB11022-3 |
| Anti-CD3 | Servicebio | GB11014 |
| Anti-CD8 | Servicebio | GB15068 |
